# Genetic Diversity and Genome-Wide Association Study of Major Ear Quantitative Traits Using High-Density SNPs in Maize

**DOI:** 10.3389/fpls.2018.00966

**Published:** 2018-07-09

**Authors:** Xiao-Mei Zhu, Xiao-Yu Shao, Yu-He Pei, Xin-Mei Guo, Jun Li, Xi-Yun Song, Mei-Ai Zhao

**Affiliations:** ^1^Key Lab of Plant Biotechnology in Universities of Shandong Province, College of Life Sciences, Qingdao Agricultural University, Qingdao, China; ^2^College of Agronomy, Qingdao Agricultural University, Qingdao, China; ^3^Key Laboratory of Qingdao Major Crop Germplasm Resource Innovation and Application, Qingdao, China

**Keywords:** maize ear traits, genetic diversity, GWAS, candidate gene, meta-QTL

## Abstract

Kernel and ear traits are key components of grain yield in maize (*Zea mays* L.). Investigation of these traits would help to develop high-yield varieties in maize. Genome-wide association study (GWAS) uses the linkage disequilibrium (LD) in the whole genome to determine the genes affecting certain phenotype. In this study, five ear traits (kernel length and width, ear length and diameter, cob diameter) were investigated across multi-environments for 2 years. Combining with the genotype obtained from Maize SNP50 chip, genetic diversity and association mapping in a set of 292 inbred lines were performed. Results showed that maize lines were clustered into seven subgroups and a total of 20 SNPs were found to be associated with ear traits significantly (*P* < 3.95E-05). The candidate genes identified by GWAS mainly encoded ubiquitin-activation enzymes (GRMZM2G015287), carotenoid cleavage dioxygenase (GRMZM2G446858), MYB-CC type transfactor, and phosphate starvation response protein 3, and they were associated with kernel length (KL) and ear diameter (ED), respectively. Moreover, two novel genes corresponding to RNA processing and fructose metabolism were found. Further, the SNPs detected by GWAS were confirmed by meta-QTL analysis. These genes and SNPs identified in the study would offer essential information for yield-related genes clone and breeding program in maize.

## Introduction

Maize (*Zea mays* L.) is one of the most significant cereal crops worldwide and plays a crucial role in sustaining food security. In addition, forage crop and industrial energy require maize as a raw material. The wide demands of maize make grain yield a major breeding target. In the past century, maize grain yield has increased eight-fold with the majority of the yield being attributed to selection and hybrid breeding (Duvick, 2005; Xiao et al., 2016). Grain yield is a quantitative trait and easily affected by environmental factors (Messmer et al., 2009; Liu et al., 2014). Kernel and ear traits, kernel length (KL), kernel width (KW), ear length (EL), ear diameter (ED), and cob diameter (CD), are all important yield components in maize (Liu et al., 2014; Zhang et al., 2014), and KL was the most effective one among them in principal component analysis (PCA). Li X. et al. (2016) also found that KL and KW were positive correlation with the single ear yield and grain yield per unit significantly. Thus, it is useful to find the genes of these traits for breeding program.

Genome-wide association study (GWAS) have been verified to be a powerful approach for identifying genes, alleles or haplotypes related to a certain agronomic traits under complex environments (Yan J.B. et al., 2011; Li X. et al., 2016), which is based on the linkage disequilibrium (LD) resulting from the association of target trait and haplotype loci. GWAS provide the opportunity to methodically analyze the genetic architecture of complex quantitative traits in many crops including maize and benefit from the high diversity and rapid LD decay in this species (Li et al., 2012). Using the 24,355 SNPs distributed in the whole genome of wheat, 38 SNPs were found to have high relationship with wheat height by GWAS analysis (Chen et al., 2015), 11 loci of which steadily expressed at least two environments. In maize, GWAS was also successfully identify numerous candidate genes controlling complex traits (Cui et al., 2016), such as plant height (Li X. et al., 2016), drought tolerance (Wang X. et al., 2016), disease resistance (Mammadov et al., 2015), stalk cell wall components (Li K. et al., 2016), ear height (Li X. et al., 2016), etc. Additionally, many QTLs about ear and kernel traits were mapped with linkage populations. Using a F_2_ population, Yang (2008) detected three consistent QTLs of maize ear diameter, which located in chromosome 2, 5, and 7 and explained 0.8, 1.5, and 0.52% of the phenotype variation, respectively. Liu et al. (2010) mapped QTLs about the number of panicles and the number of row grains with 239 recombinant intersections of Mo17 × Huangzaosi by composite interval mapping (CIM). Li et al. (2009) detected QTLs of KL on chromosome 1, 3, and 6, respectively, with a F_2:3_ population created from Qi 319 and Huangzaosi. Six QTLs identified by Liu (2013) were individually accounted for 1.18–12.92% of the phenotypic variation. Wang (2015) detected one QTL-qKL9 for KL by using 263 single plants of BC_2_F_2_ population, which explained 14.38% of the phenotypic variation. Qin et al. (2015) detected a total of seven QTLs distributing on chromosome 1, 4, 7, and 10, respectively. And the main effect QTL for KL, named as *qklen1*, was mapped on the physical location of 210–212 Mb on chromosome 1 using the average value of multi-environments. Qin et al. (2015) identified 22 QTLs about KW distributed on chromosomes 1, 2, 3, 4, 5, 6, 9, and 10, respectively, and among them, one QTL on chromosome 10 was further mapped on the physical location of 147 Mb. Also, another four QTLs for KW was detected on each of chromosome 4, 6, 9, and 10 using average value of multi-environments, and named as *qkwid4, qkwid6, qkwid9 and qkwid10*, respectively (Qin et al., 2015). These identified QTLs and genes were helpful for studying the mechanism of yield-related traits. However, the above studies often use biparental mapping populations, which could not reveal the genetic variation of broader genetic back-ground.

Recently, some kernel related genes had been cloned. With Zheng 58 as the plant material, Xia et al. (2016) obtained a gene *ZmMADS-RIN* using the homologous cloning method, which has sufficient homology with the gene *OsMADS6* related to kernels development in rice. Based on the yield-related gene *OsGW2* in rice, Kong et al. (2014) cloned a maize homologous gene *ZmGW2-1*, which encoded E3 ubiquitin ligase protein and probably regulated the development of ear. A CLAVATA receptor protein locus on chromosome 4 was cloned, and its mutation led to the increase of meristem and ear row number (Bommert et al., 2013). Using a F_2_ population constructed by a near isogenic line, a 3 kb intergenic region at downstream of *Unbranched3* (*UB3*) gene on chromosome 4 was found, which was responsible for the quantitative variation in kernel row number (KRN) by regulating *UB3* expression (Liu L. et al., 2015). According to a key kernel size-related gene (*OsGS5*) in rice, a 981 bp gene segment *ZmGS5* was obtained using homologous cloning method, which encoded protein sequence that belonged to serine carboxypeptidase in maize (Li et al., 2014). And this sequence was as highly as 75% homologous to the protein sequence encoded by *OsGS 5* in rice. The results of bioinformatics analysis carried out by Tian et al. (2014) showed that the gene *GRMZM2G070323* on chromosome 1 and *GRMZM2G148539* on chromosome 5 in maize were homologous with a kernel length-related gene (*OsPPKL*) in rice. At present, most of yield-related genes of maize come from the homology genes in rice, so the study of GWAS and QTL mapping for maize yield-related traits is imperative.

In this study, the phenotype of five ear traits for an association mapping panel consisting of 292 maize inbred lines were collected in 2015 and 2016. Then, QTLs associated with the five traits were identified with GWAS method, and candidate genes were also predicted. This study would provide useful insights into the genetic basis of related traits, and supply molecular tools for improving kernel size and grain yield in maize.

## Materials and Methods

### Materials

A total of 292 maize inbred lines (**Supplementary Table S2**) were analyzed in the study, which were derived from four subgroups of China, Reid, Lvdahonggu, P group, and Sipingtou, as well as some tropical lines and sweet-waxy maize. All the materials were collected or bred by the maize molecular breeding team, Qingdao Agriculture University, China and were commonly used in maize breeding program.

### Experimental Design and Phenotyping

The 292 maize inbred lines were grown at three locations of China in 2 years, which were Qingzhou, Shandong Province in 2015 and 2016 (QZ15 and QZ16), Luoyang, Henan Province 2015 (LY15), and Jiaozhou, Shandong Province 2016 (JZ16). Experiment was arranged in a randomized complete block design with three replications, and each inbred line was grown in a single row with 15 plants, 3 m in length, 0.6 m between adjacent rows, and 0.2 m between adjacent plants. The measure method of KL and KW were as described in Jiang et al. (2015). The field management followed normal agricultural practices.

When harvest, five well-developed ears in the middle of each row were selected so as to minimize the boundary effect. A digital caliper (Guilin Guanglu Measuring Instrument Co., Ltd., CHINA) was used to measure KL, KW, EL, EW, and CD, in which EW and CD were measured at the middle of each ear and cob, KL and KW were measured with ten mixed and randomly selected kernels from each inbred line. To ensure accuracy, the data of each trait were determined with the average value of three replications.

### Statistical Analysis of Phenotypic Data

SPSS20 statistical software (Armonk, NY, United States: IBM Corp) was used to calculate the phenotypic data, including Normal analysis of each trait and Pearson correlation analysis between traits and environments.

The broad-sense heritability (*h*^2^) was calculated using the following formula:

$$H^{2}=\frac{V_{\text{G}}}{V_{\text{p}}}\times 100\%=\frac{V_{\text{G}}}{V_{\text{G}}+V_{\text{E}}}\times 100\%$$

Where, *V*_P_ is phenotype variation, *V*_G_ is genetic variation, *V*_E_ represents environmental variance. Basing on the formula, the smaller the variance of the environment was, the higher ratio the genetic variance in the phenotypic variance accounted for, thus the genetic variation is mostly inherited, and vice versa.

### DNA Extraction and SNP Genotyping

Genomic DNA was extracted from the tender leaves at the six-leaf growth stage with the modified cetyltrimethylammonium bromide (CTAB) method (Chen and Ronald, 1999). A total of 56,110 SNPs were selected from the whole maize genome and anchored on a maizeSNP50 DNA chip, and then each inbred line was genotyped with the chip from Pioneer Dupont (United States). After the SNPs with missing rate >20% and heterozygosity >20% were excluded, 35,355 SNPs was kept to be analyzed further. Then, some SNPs with minor allele frequency (MAF) <0.05 also were excluded through genetic diversity analysis of maize population, and only 25,331 SNPs were left for GWAS analysis. The number of alleles and allele frequency of each SNP locus was calculated with PowerMarker V3.25 software^1^ (Liu and Muse, 2005) .

### Molecular Diversity, Linkage Disequilibrium and Population Structure Analysis

Genetic distance of the 292 inbred lines and LD of each chromosome were calculated with Cladogram and LD functions of Tassel 5.2.31^2^ (Bradbury et al., 2007), respectively, and the neighbor-joining (NJ) cluster map constructed. LD level and its decay rate between each pair of SNPs on each chromosome was analyzed with the squared of Pearson correlation coefficient (*r*^2^). The calculation result was imported into Excel to create the LD decay plot.

The molecular markers used for subgroups division by the Structure V2.3.4 software^3^ (Pritchard et al., 2000; Evanno et al., 2005) were selected bases on the distance between the markers of *r*^2^ = 0.1. Bayesian cluster analysis of 292 maize inbred lines was carried out using the selected 1361 independent SNPs. Then, the reasonable subgroups number (*K*) of the population was inferred according to ln*P* (D) value in Structure V2.3.4. With the optimum *K*-values ranging from 1 to 10, the strong Markov Chain Monte Carlo (MCMC) after the non-repeated iteration was set to 10000 times at the beginning, and then set to 50000 times with the number of iterations set at 7. The probability of each inbred line grouped into a subgroup to determine the genetic composition of materials.

### GWAS Analysis

Fixed and random model Circulating Probability Unification (FarmCPU) (Liu, 2015) was adapted for analyzing large data and calculation speed was quick. With this model, an iterative usage of fixed and random effects for powerful and efficient GWAS was developed to solve the mixed problem of false positive and false negative SNPs in MLM. Then, a total of 25331 SNPs were used for GWAS, with the genome-wide threshold of *P* = 1/total number of SNPs = 3.95E-05 (Wang et al., 2015; Bai et al., 2016; Ma et al., 2016). Further, to ensure the GWAS results with FarmCPU model, we did GWAS analysis using the compressed mixed linear model (CMLM) in GAPIT package (Lipka et al., 2012). The CMLM is a compression and optimization model based on MLM in Tassel.

### Candidate Genes Mining

Based on the SNP locus which were significantly association with target traits, the genome sequence of the maize line B73 was used as the reference genome for selecting candidate gene (Schnable et al., 2009; Liu et al., 2016). The genes corresponding to each SNP locus were checked using the molecular marker database in MaizeGDB^4^ according to the physical positions of the SNPs. Then, the functional annotations of candidate genes were predicted in NCBI^5^. The significant association regions were scanned for putative genes using IGV downloaded^6^, and LD analysis of the linkage SNPs in 310-kb window were conducted using Haploview v.4.2^7^.

### Integration of Meta-QTL

Now many QTLs have been reported, but most of them are different due to different mapping populations, different analysis methods, and different environmental conditions, thus resulting in the problems of oversizing or overlapping of QTL position. In this study, we used the meta-analysis function in software BioMercator V4.2, to integrate the many QTLs related to ear traits into the IBM2 2008 Neighbors Map. Thus, many information of QTLs on maize ear traits were collected from the main literatures published from 2007 to 2016 basing on China National Knowledge Infrastructure (CNKI^8^ and NCBI, which included the size and type of the mapping population, the markers’ genetic distance, mapping function, and the position, LOD value, contribution rate, and confidence intervals of QTLs. Because the maximum likelihood value, confidence interval, and contribution rate are important information of QTL (Okuda et al., 2007), the 95% confidence interval of each QTL is inferred from the two equations below before it was introduced into the IBM2 2008 Neighbors Map.

1 $$CI=530/(N^{*}R^{2})$$

2 $$CI=163/(N^{*}R^{2})$$

Where, CI is the confidence interval, *N* represents the mapping population size, *R*^2^ represents the contribution ratio. And Equation 1 is suitable for backcross and F_2_ mapping populations, and Equation 2 is suitable for the recombinant inbred lines (Qi et al., 2011).

So, the high-density genetic linkage map IBM2 2008 Neighbors was obtained. The specific position on the map successively contained 1, 2, 3, 4, and N “real” QTL(s) in five models of QTL given by the simulation operation, and the optimal model was judged by the value of the minimum Akaike-type criteria value (AIC) (Goffinet and Gerber, 2000). And the initial QTLs used for mata-QTL was not less than three.

## Results

### Genetic Diversity, Linkage Disequilibrium and Population Structure Analysis

**FIGURE 1:**
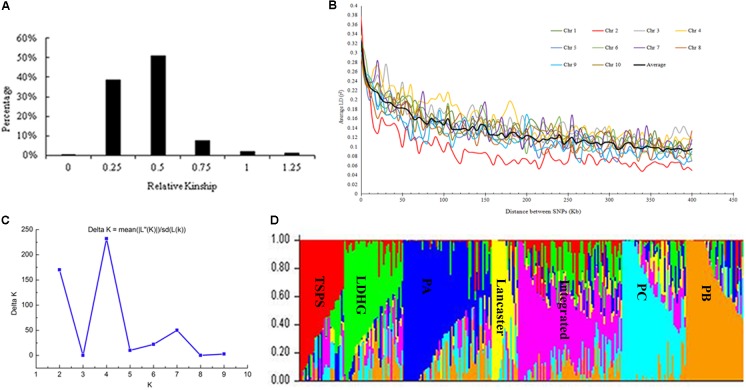
Analysis of genetic diversity. **(A)** Distribution of pairwise relative kinship for 292 maize inbred lines calculated using 25331 filtered SNPs. **(B)** Whole-genome LD in the entire panel based on 292 maize inbred lines. **(C)** Δ*K*-value of 292 inbred lines based on 1362 SNPs. **(D)** The Bayes cluster plot of 292 maize inbred lines when *K* = 7.

Based on the 25331 SNPs for GWAS, we made relationship diagram among 292 maize inbred lines (**Figure 1A**). And 96.75% of the relationship values between two lines ranged between 0.25 and 0.75, and only 0.06% of them was zero, 3.19% >1, and 38.48% <0.5, which mean that most of the materials are relatively relevant to each other, with a few irrelevant.

The LD level of the whole genome of inbred lines was estimated using 25331 SNPs. Results showed that LD decayed differently in ten chromosomes, with chromosome 2 had the most rapid decay rate and chromosome 4 had the slowest. At a cut-off value of *r*^2^ = 0.1, the averaged LD decay distance of 292 maize inbred lines was approximately 310 kb (**Figure 1B**).

**FIGURE 2:**
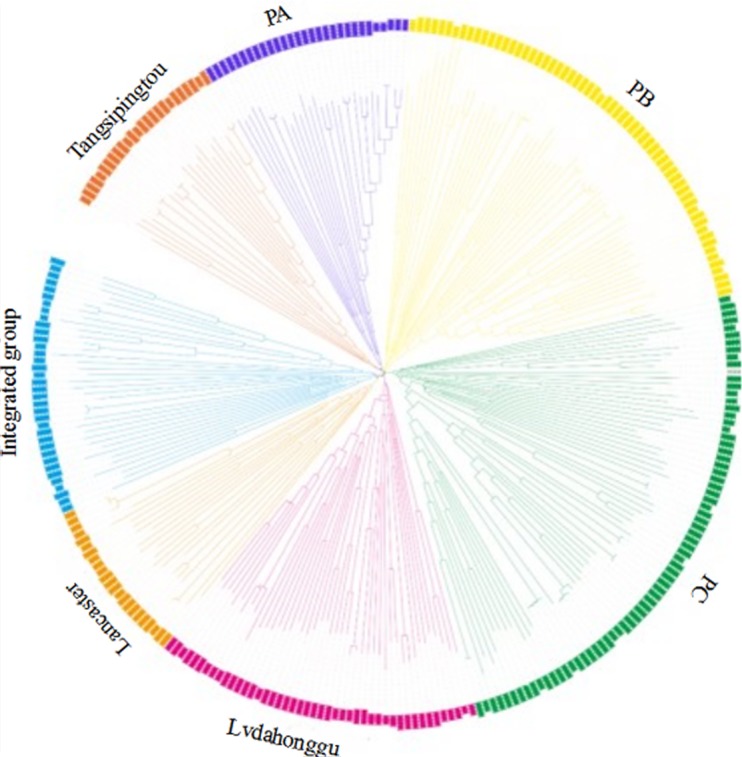
Neighbor-joining (NJ) tree for the 292 maize inbred lines based on 25331 SNPs analysis data.

The genetic distance between 292 maize inbred materials was calculated by Tassel 5.2.31, and a neighbor-joining clustering graph was constructed (**Figure 2**). The entire materials were divided into seven groups, namely, Tangsipingtou, PA, PB, PC, Lvdahonggu, Lancaster, and an integrated groups. Tangsipingtou group mainly consists of inbred lines such as Chang 7-2, Lx 9801, H 21 as well as hybrids selected from Chang 7-2 hybridizing with other materials in this study. The PA group tends to Reid, including Zheng 58, Ye 478 etc. PB group mainly includes Ex, Qi 319, P 138, Qi 318, X 178 and so on. PC group tends to BSSS, mainly including B73. Lvdahonggu group mainly contains E28, Dan 340 and other materials. Most of the inbred lines were clustered into their corresponding subgroup, and the tropical lines and sweet-waxy maize derived from Philippines and Mexico were clustered into the integrated group. However, a few inbred lines belonging to Lancaster subgroup, such as Qi 205 and Ji 846, were diffused in other subgroups.

The genetic diversity results analyzed with the Structure software are the same as that of NJ clustering. When *K* = 7, the 292 inbred lines could be grouped into seven large subgroups (**Figures 1C,D**).

### Phenotype Statistics

**FIGURE 3:**
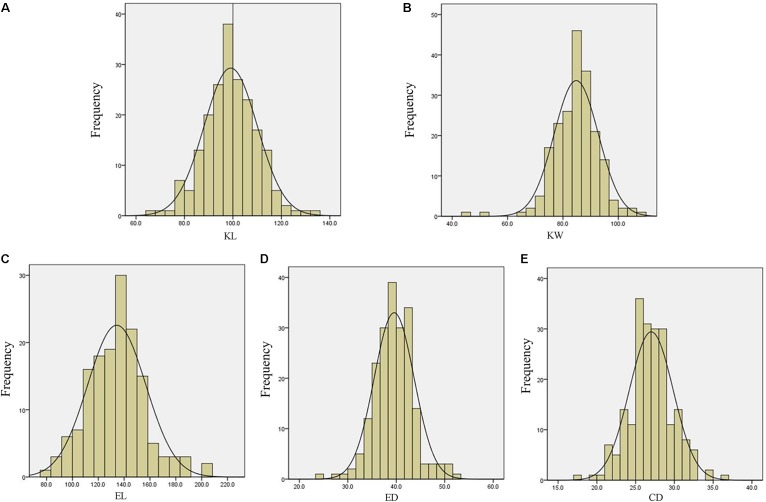
Phenotypic distribution for measured traits of Luoyang in 2015. **(A)** KL, kernel length; **(B)** KW, kernel width; **(C)** EL, ear length; **(D)** ED, ear diameter; **(E)** CD, cob diameter.

The descriptive statistics for ear and kernel traits under three natural environments in 2 years are presented in **Table 1**. Abundant and large variation of the five traits, KL, KW, EL, ED, and CD, was observed in each location. But the variation values of each trait were different at different environments. For example, the variation for the KL in LY15 ranged from 65.04 to 135.96 mm (mean ±*SD* = 98.54 ± 10.40 mm), but it ranged from 67.30 to 117.95 mm (92.71 ± 9.04 mm) in JZ16. The *H*^2^ of the five traits was relatively high, ranging from 68.86 for EL to 85.88% for ED (**Table 1**), indicating that a large portion of phenotypic variance for ear and grain traits could be attributed to genotypic effects. All the phenotypic data of every trait follow Normal distribution, as the absolute values of kurtosis and skewness among these environments were less than 1, thus they were suitable for QTL mapping (**Figure 3**).

**Table 1:** Descriptive statistics and broad-sense heritability (*H*^2^) for five traits including kernel length (KL), kernel width (KW), ear length (EL), ear diameter (ED), and cob diameter (CD) among 292 accessions across four environments.

Trait	Environment	Minimum	Maximum	Mean ± SD	CV (%)	Skewness	Kurtosis	*H*^2^(%)
KL	2015LY	65.04	135.96	98.54 ± 10.40	10.55	0.049	0.515	79.51
	2015QZ	65.62	121.98	92.20 ± 9.36	10.15	0.196	0.503	
	2016JZ	67.30	117.95	92.71 ± 9.04	9.75	−0.004	−0.093	
	2016QZ	69.61	121.01	95.57 ± 9.21	9.64	−0.053	−0.313	
KW	2015LY	64.21	105.20	85.26 ± 6.59	7.73	−0.093	0.146	88.74
	2015QZ	66.17	109.81	86.21 ± 7.67	8.90	0.173	0.211	
	2016JZ	60.24	101.45	81.58 ± 7.33	8.99	−0.157	0.569	
	2016QZ	55.42	103.34	83.70 ± 6.74	8.05	−0.237	0.845	
EL	2015LY	79.00	205.00	133.60 ± 22.52	16.86	0.357	0.567	68.86
	2015QZ	79.80	181.40	111.37 ± 20.50	18.41	−1.362	0.433	
ED	2015LY	24.32	51.89	39.54 ± 4.16	10.52	−0.029	0.979	85.88
	2015QZ	25.10	52.22	40.16 ± 4.13	10.28	−0.33	0.594	
	2016JZ	26.93	49.78	37.16 ± 3.71	9.98	0.096	0.386	
	2016QZ	26.95	46.86	38.16 ± 3.50	9.17	−0.326	−0.124	
CD	2015LY	17.20	36.47	27.03 ± 2.84	10.51	−0.055	0.504	85.18
	2015QZ	15.60	35.48	25.71 ± 3.01	11.71	0.004	0.719	
	2016JZ	14.87	34.02	25.81 ± 2.84	11.00	0.026	0.219	
	2016QZ	17.77	33.11	26.27 ± 2.80	10.66	−0.13	0.011	

*SD, standard deviation; CV, coefficient of variation.*

### Correlation Analysis

To verify the accuracy and consistency of the results, ANOVA was conducted to reveal the significant correlation among the five ear traits in 2 years (**Table 2**). Among all the positive correlations in 2015, the highest value was found between ED and CD, while the lowest value was between ED and KW. In 2016, significantly positive correlations were obtained between all traits except KL and CD in Jiaozhou (**Supplementary Table S1**).

**Table 2:** Phenotypic correlation coefficient estimates for each trait in Luoyang, 2015 (above diagonal) and Qingzhou, 2015 (below diagonal).

Trait	KL	KW	EL	ED	CD
KL		0.090^ns^	0.295^∗∗^	0.765^∗∗^	0.274^∗∗^
KW	0.204^∗∗^		0.133^ns^	0.183^∗∗^	0.223^∗∗^
EL	−0.104^ns^	0.018^ns^		0.442^∗∗^	0.341^∗∗^
ED	0.529^∗∗^	0.341^∗∗^	0.103^ns^		0.738^∗∗^
CD	0.255^∗∗^	0.169^∗∗^	−0.119^∗^	0.770^∗∗^	

*KL, kernel length; KW, kernel width; EL, ear length; ED, ear diameter; CD, cob diameter. ^∗∗^, significant at p < 0.01, ^∗^, significant at p < 0.05; ns, not significant.*

### GWAS Analysis

**FIGURE 4:**
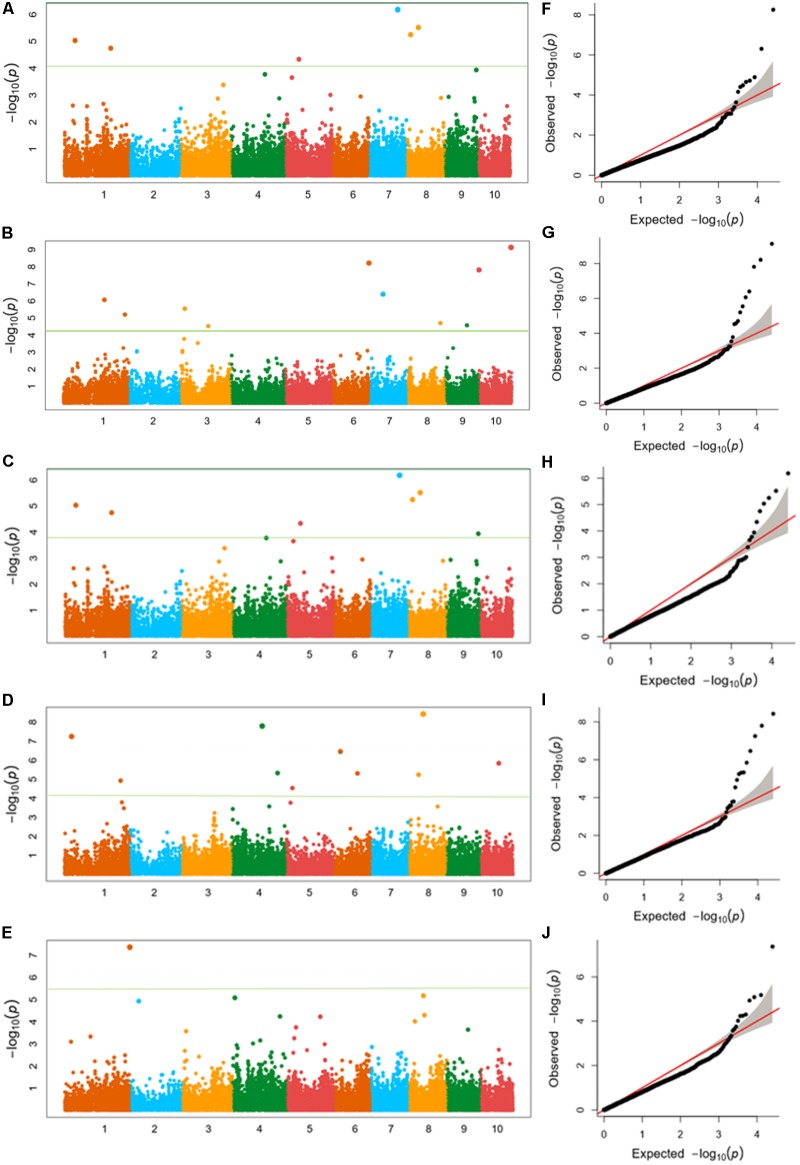
**(A–E)** Manhattan plots of **(A)** kernel length, **(B)** kernel width, **(C)** ear length, **(D)** ear diameter, **(E)** cob diameter for the 10 chromosomes carrying the significant markers detected by FarmCPU in Luoyang, 2015; **(F–J)** Quantile–Quantile (Q–Q) plots of the five traits in the same order with Manhattan plots showing expected null distribution of *p*-value assuming no associations, represented as solid red line; distribution of *p*-value observed.

Using FarmCPU, the phenotype data of five ear traits and the genotype data of 25331 SNPs were analyzed for GWAS. Quantile–quantile (Q–Q) plots implied that the population structure and kinship relationship were well controlled in the GWAS for each trait. The horizontal and the verticle axises show the values of -lg (Transformed expected *P*-value) and -lg (Transformed observed *P*-value), respectively (**Figure 4** and **Supplementary Figures S1**–**S3**).

Twenty SNPs, significantly associated with the five ear-related traits, have been detected and distributed on eight chromosomes of maize (**Table 3**). Among them, four SNPs, associated with KL dispersed on chromosome 3, 6, and 7 (2), were detected. Especially, the SNP locus PZE_107042407 on bin7.02 was detected both in JZ16 and LY15. For KW, three SNPs were detected only in LY15 and distributed on chromosomes 6 and 10 (2), respectively. Additional, one SNP locus associated with EL, seven loci associated with ED, and five associated with CD were also identified. Among all the SNPs related to ear traits in this study, the one related to CD on chromosome 1 was the most significant among them. All the details of SNPs and candidate genes were shown in **Supplementary Table S3**.

**Table 3:** Most significant marker loci associated with five traits over 2 years in three locations.

Trait	SNP ID	Chr	Bin	Alleles	*P*-value	Additive effect	Env
KL	PZE_103141524	3	3.07	C/T	2.31E-14	−3.36	QZ16
	PZE_106099248	6	6.04	C/T	5.52E-09	2.35	LY15
	PZE_107042407	7	7.02	A/G	9.93E-06	2.79	JZ16
	PZE_107042407	7	7.02	A/G	2.23E-05	2.48	LY15
KW	PZE_110105598	10	10.07	C/T	7.38E-10	−2.02	LY15
	SYN4309	6	6.07	C/G	6.09E-09	−1.91	LY15
	PZE_110000228	10	10.00	C/G	1.54E-08	−3.95	LY15
EL	PZE_107081628	7	7.03	A/G	6.61E-07	9.31	LY15
ED	PZE_110044605	10	10.03	G/T	1.29E-10	−1.07	LY15
	PZE_108042082	8	8.03	A/G	3.75E-09	−1.71	LY15
	PZE_104069344	4	4.05	T/C	1.61E-08	−1.27	LY15
	PZE_103171163	3	3.09	A/G	2.07E-07	1.00	JZ16
	PZE_104124003	4	4.09	A/G	4.67E-06	0.67	LY15
	PZE_108042082	8	8.03	A/G	6.45E-05	−1.75	QZ16
	PZE_105059330	5	5.03	A/G	9.97E-05	−0.76	QZ15
CD	SYN13476	1	1.08	A/C	4.11E-08	−0.79	QZ16
	PZE_101255159	1	1.12	C/T	4.31E-08	−0.72	LY15
	PZE_101116177	1	1.05	A/C	3.86E-06	−0.72	QZ15
	PZE_108042082	8	8.03	A/G	6.64E-06	−0.85	LY15
	PZE_101223850	1	1.10	C/T	6.16E-05	−0.47	QZ15

*Chr, chromosome. Traits. KL, kernel length; KW, kernel width; EL, ear length; ED, ear diameter; CD, cob diameter. Environment. LY15 and QZ15 represent Luoyang and Qingzhou in 2015, respectively; JZ16 and QZ16 represent Jiaozhou and Qingzhou in 2016, respectively.*

**FIGURE 5:**
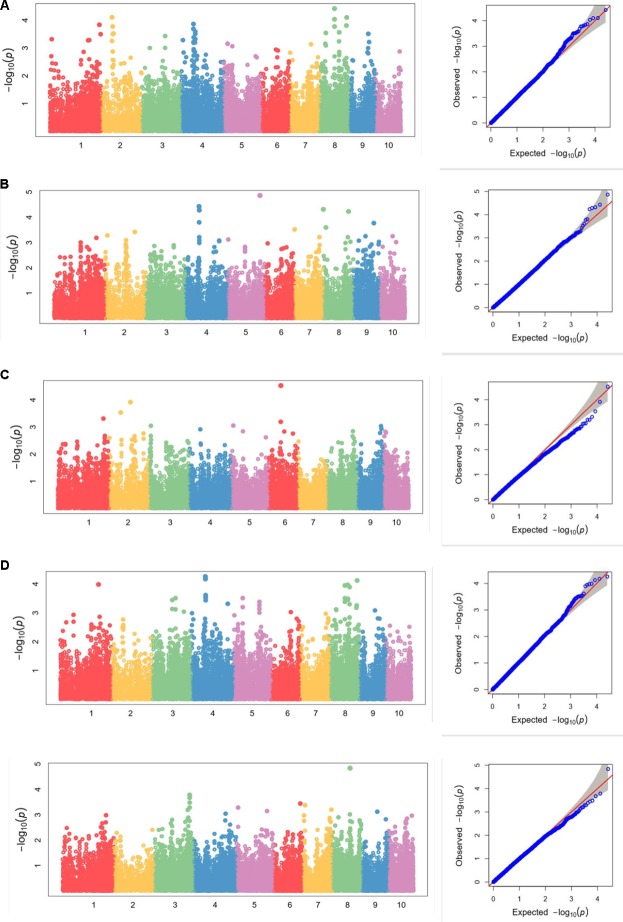
**(A–E)** Manhattan plots and Q-Q plots of **(A)** kernel length, **(B)** kernel width, **(C)** ear length, **(D)** ear diameter, **(E)** cob diameter resulted by GAPIT in Luoyang, 2015.

**FIGURE 6:**
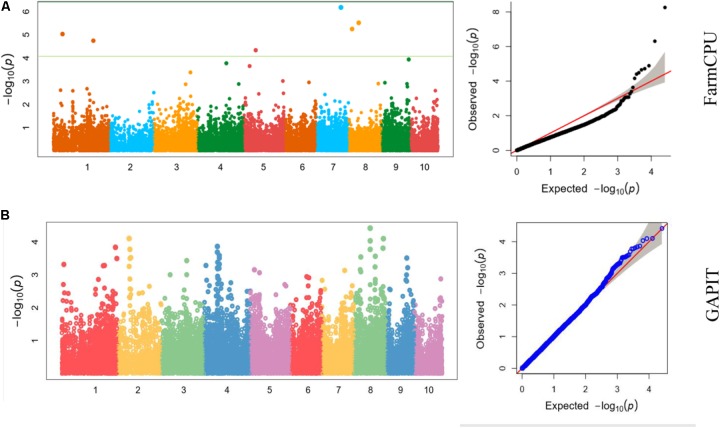
Manhattan plots and Q-Q plots of kernel length in Luoyang, 2015 resulted by **(A)** FarmCPU and **(B)** GAPIT.

We also analyzed the five traits using CMLM model in GAPIT package (**Figure 5**). In the Q-Q plots of association studies, the signal above the Bonferroni correction line by FarmCPU was better than that by CMLM model in GAPIT, which suggested that candidate genes were difficult to be distinguished from the background noise by CMLM. Namely, CMLM reduces the detection efficiency of the associated sites with known candidate genes when compared with FarmCPU (**Figure 6**). In our study, and the significantly association loci decreased to 26 when the CMLM was employed.

### Candidate Genes Predicting

**FIGURE 7:**
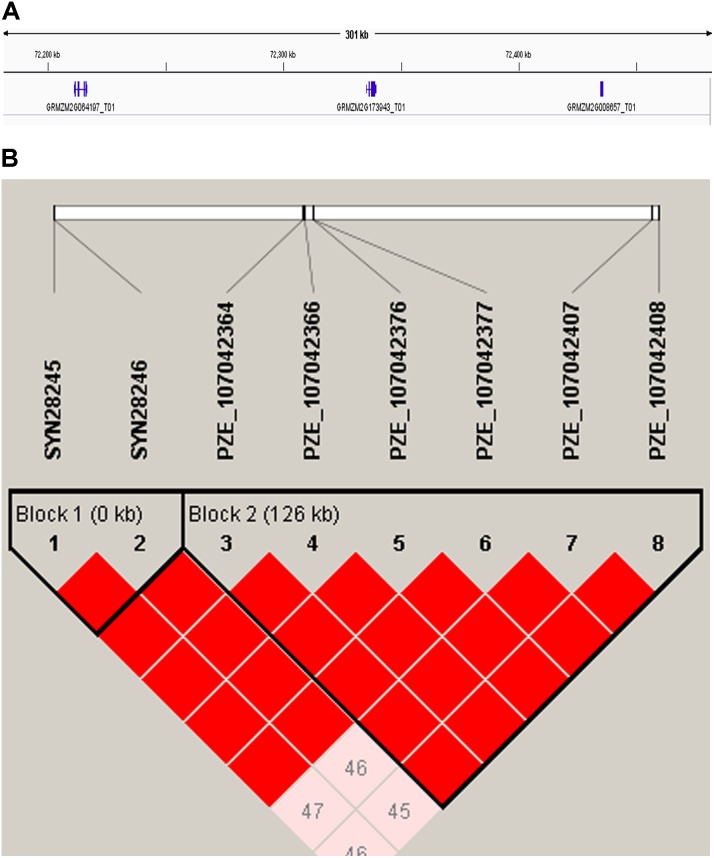
**(A)** 310-kb association region of gene GRMZM2G173943 on chromosome 7. **(B)** The linkage disequilibrium blocks of the major SNP (PZE_107042407) within 310-kb window.

Candidate genes containing SNPs associated with the five ear-related traits were identified, and their function were predicted basing on MaizeGDB and NCBI (**Table 4**). SNP locus, PZE_107042407, which was detected in two environments of JZ16 and LY15 simultaneously, was located within the interval of gene GRMZM2G173943, whose functional annotation is MYB-CC type transfactor. Reported genes, *Arabidopsis AtPHR1* and algal *PSR1*, are members of MYB-CC gene family and regulated phosphorous hunger signal pathway (Moseley et al., 2006; Li, 2007; Bournier et al., 2013). Besides, a gene related to ED (LOC103626417), responded to protein phosphoric acid hunger was discovered in this study. Three SNPs associated with KW were located within the intervals of gene GRMZM2G124502, GRMZM2G092475, and GRMZM2G057441, respectively, and the three genes are related to encoding SWIB complex BAF60b domain-containing protein, probable sodium/metabolite co-transporter BASS4, chloroplastic and ubiquitin-activating enzyme E1 2, respectively. What’s more, four candidate genes related to ubiquitin, GRMZM2G057441, GRMZM2G015287, GRMZM2G018798, and LOC100381748 were screened. Among them, GRMZM2G015287 correlated with ED and CD was detected in LY15 and QZ16 simultaneously. The genes located at the association regions flanked by three significant SNPs were also identified within the estimated 310-kb window (**Table 5**). Two linkage genes at chr: 72.21–72.53 Mb were identified in the same LD block with PZE_107042407 (**Figure 7**).

**Table 4:** Candidate genes for each significant SNP associated with traits and theirs encoding products.

Trait	Chr	SNP physical position (Mb)	Gene ID	Encoding
KL	3	197085927	GRMZM2G446858	Carotenoid cleavage dioxygenase
	6	152940683	GRMZM2G067198	Hypothetical protein
	7	72,337,865	GRMZM2G173943	MYB-CC type transfactor
	7	72,337,865	GRMZM2G173943	MYB-CC type transfactor
KW	10	146673417	GRMZM2G124502	SWIB complex BAF60b domain-containing protein
	6	165488800	GRMZM2G092475	Probable sodium/metabolite cotransporter BASS4,chloroplastic
	10	1153678	GRMZM2G057441	Ubiquitin-activating enzyme E1 2
EL	7	136607023	GRMZM2G074386	Proteasome subunit beta type-1
ED	10	85027699	GRMZM2G126007	UPF0481 protein At3g47200
	8	67245292	GRMZM2G015287	Ubiquitin-conjugating enzyme E2 N
	4	206956947	LOC100381748	Ubiquitin carboxyl-terminal hydrolase 13
	4	137099594	GRMZM2G179810	Adenine phosphoribosyltransferase 2
	3	218272659	GRMZM2G156126	Alpha/beta-hydrolases superfamily protein
			GRMZM2G156158	P-loop containing nucleoside triphosphate hydrolase superfamily protein
	1	275012730	GRMZM2G018798	E3 ubiquitin protein ligase DRIP2
			GRMZM2G398608	Peptide chain release factor PrfB1, chloroplastic
	5	58165601	LOC103626417	Protein PHOSPHATE STARVATION RESPONSE 3
CD	1	249380954	GRMZM2G103843	Fructokinase-like 2, chloroplastic
	1	298903090	GRMZM2G084176	Putative pentatricopeptide repeat-containing protein
	1	139605674	GRMZM2G014872	SEC1 family transport protein SLY1
	8	67245292	GRMZM2G015287	Ubiquitin-conjugating enzyme E2 N
	1	275011999	GRMZM2G018798	E3 ubiquitin protein ligase DRIP2
			GRMZM2G398608	Peptide chain release factor PrfB1, chloroplastic

**Table 5:** Candidate genes scanned within a approximately 310 kb extended region for three significant SNPs.

SNP	Bin	Mb	Gene ID	Description
PZE_107042407	7.02	72.21–72.53	GRMZM2G064197	myb family transcription factor-related protein
			GRMZM2G173943	MYB-CC type transfactor
			GRMZM2G008657	Putative uncharacterized protein DDB_G0287975
PZE_108042082	8.03	67.10–67.40	GRMZM2G119316	HSP40/DnaJ peptide-binding protein
			GRMZM2G015287	Ubiquitin-conjugating enzyme E2 N
PZE_110105598	10.07	146.51–146.81	GRMZM2G28492	Zinc finger C-x8-C-x5-C-x3-H type family protein
			GRMZM2G86949	Auxin response factor 11
			GRMZM2G124502	SWIB complex BAF60b domain-containing protein
			GRMZM2G124495	GLK52 transfactor
			GRMZM2G124476	Zinc finger C-x8-C-x5-C-x3-H type family protein;
			GRMZM2G124466	putative plastid-lipid-associated protein 13 chloroplastic
			GRMZM2G125149	Trafficking protein particle complex subunit 3
			GRMZM2G423861	Kinesin-like protein KIN-14I
			GRMZM2G125083	N-acylphosphatidylethanolamine synthase
			GRMZM2G405662	Heavy metal transport/detoxification superfamily protein
			GRMZM2G107620	Regulatory protein RecX family protein
			GRMZM2G107557	Plant cysteine oxidase 3

*C.I., confidence interval.*

### Meta-QTL Analysis

**FIGURE 8:**
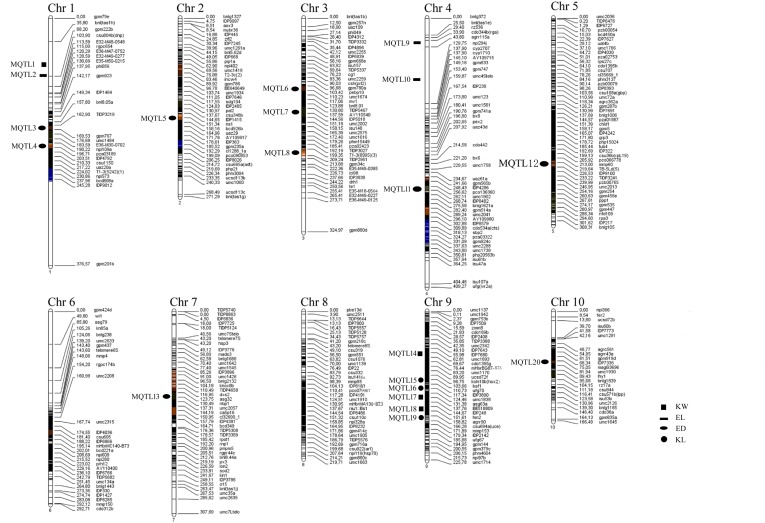
“Consensus” QTL map integrated for ear and kernel traits in maize.

Information of 293 QTLs related to the panicle traits (**Table 6**) were collected and used to construct the consistency map (**Figure 8**). In the meta-analysis, the model with the minimum AIC value was chosen as the optimal one, then 20 meta-QTLs were obtained according to the selection criteria of at least three initial QTLs existing within one location. These meta-QTLs are mainly distributed on chromosomes 1(4), 3 (3), 4 (3), and 9 (5), respectively, with confidence intervals ranging from 4.2 to 15.13 cM (**Table 7**). Then, these meta-QTLs and the SNPs associated with ear traits in this study was mapped according to the physical distance of SNP on both sides of MQTL tag, Results showed that five of the detected SNPs was located within the intervals of these meta-QTLs, such as locus PZE_101048890 in MQTL4 interval, PZE_103171163 in MQTL8, SYNGENTA6857 in MQTL12, and PZE_110067406 and PZE_110061773 were both in MQTL20 interval. These results further i verified the accuracy of the SNP loci related to ear traits in this study.

**Table 6:** Summary of the QTLs of ear traits in maize (2007–2016).

Parents	Pop size	Type	Method	Reference
Dan232, N04	258	F_9_	CIM	Li et al., 2011
Dan232, N04	220	BC_2_F_2_	CIM	Li et al., 2007
PB260, PB266	149	F_2:3_	CIM	Mendesmoreira et al., 2015
Mc, V671	270	F_2:3_	CIM	Liu et al., 2014
SK, Zheng58	204	F_6_	CIM	Raihan et al., 2016
Si287, Si144	187	F_3_	CIM	Sun et al., 2013
Nongxi928	161	ILs	ICIM	Zhu et al., 2012
L26, 095	186	F_2_	CIM	Dai et al., 2009
092, Jiao51	400	F_2_	IM	Lü et al., 2008
Nongdan5	79	DH	CIM	Zhang et al., 2009
Nongxi110, 53	95	BC_2_F_2_	CIM	Hu et al., 2010
Ye478, Nan340	397	F_2:3_	CIM	Yang, 2008
Zong387-1	294	F_9:10_	CIM	Bai, 2014

**Table 7:** Information of position of Meta-QTL by meta-analysis for ear and kernel traits in maize.

Meta-QTL	Chr	Position	C.I.(cm)	Left marker	Right marker	Triat
MQTL1	1	65.08	63.12–67.05	mmp172	pco135045	KW
MQTL2	1	72.95	69.68–76.22	IDP637	geb7	KL
MQTL3	1	167.36	164.58–170.13	cdo1081a	AY108650	ED
MQTL4	1	195.34	192.07–198.62	npi423	agrr92a	ED
MQTL5	2	151.09	146.21–155.97	les10	umc1003	ED
MQTL6	3	107.31	103.77–110.84	bnlg1957	uaz37	ED
MQTL7	3	143.05	135.49–150.62	IDP4818	IDP7565	ED
MQTL8	3	204.96	199.26–210.66	T1-39(8995) (3)	IDP9094	ED
MQTL9	4	36.65	34.65–38.73	cdo344b	bx6	EL
MQTL10	4	54.6	37.5–63.1	umc2276	umc1738	EL
MQTL11	4	260.33	255.41–265.24	pco136360	umc49j	ED
MQTL12	5	221.53	216.54–226.52	pza03049	umc2527	ED
MQTL13	7	129.39	123.89–134.88	agrc261	npi47b	ED
MQTL14	9	58.35	51.34–60.64	glk1	gpm289c	KW
MQTL15	9	87.44	86.12–88.76	umc1921	asn4	KL
MQTL16	9	92.6	91.52–93.69	mHbrMT307_Mo17	umc1494	KL
MQTL17	9	114.31	100.6–120.4	TIDP2493	npi97b	KW
MQTL18	9	137.7	131.45–143.95	PZE-109109569	SYN8851	KW
MQTL19	9	148.75	139.45–158.05	PUT-163A-94472707-4860	SYN5732	KL
MQTL20	10	77.44	75.34–79.54	cdo482	bnl17.08	ED

*C.I., confidence interval.*

## Discussion

Natural germplasm with a broad genetic base could be a potential resource for improving yield (Wang H. et al., 2016). Genetic diversity analysis of the germplasm available provides key information on heterosis exploitation and breeding strategies, especially in maize hybrid breeding. In the present study, a panel consisting of 292 inbred lines representing temperate germplasm from Huang-Huai-Hai region were separated into seven subgroups, Tangsipingtou, Lvdahonggu, PA, PB, PC, Lancaster and an integrated group based on NJ cluster analysis, which were consistent with the results of UPGMA tree analyzed with genetic distance by Rogers (1972). The integrated group mainly included the tropical lines from Mexico, and the other six were consistent with the subgroups of maize germplasm in China (Liu C.L. et al., 2015). In addition, because LD patterns of population structure are crucial for association mapping and selection of candidate genes (Liu et al., 2014), LD of the 292 inbred lines was also analyzed, with the average LD decay distance of about 310 kb, which was similar to the 391 kb of 367 inbred lines in Wu et al. (2014), but lower than the 643 bp in 240 temperate inbred lines (Liu et al., 2014) and the 0.50–0.75 Mb in 362 Southwest lines of China (Zhang et al., 2016). The lower LD of the materials in this study was due to that most of the 292 inbred lines were temperate germplasm, while it was still lower than that of other temperate population suggested that the these materials have some excellent genes related to plant resistance (Lu et al., 2011).

Usually, a lot of QTLs for a certain trait have been detected with different populations in different environments, and most of them are not consistence with each other. In previous studies, Yang et al. (2016) detected six QTLs for KL and KW, with a minimum physical distance of 0.72 Mb between chr. 2-11541 and chr. 2-12258 on chromosome 2. Liu et al. (2014) detected 83 QTLs for KL and KW with a minimum physical interval of 1.37 Mb and average of 17.30 Mb between umc1165 and umc1265 on chromosome 2. Li et al. (2013) detected three QTLs for KL at bin 1.07, 4.08, and 9.03, respectively, and seven QTLs for KW at bin 1.04, 1.11, 2.07, 3.07, 4.03, 4.05, and 10.07, respectively. Previous reports also showed that bin 4.05 and bin 10.03 were important genomic regions for controlling maize yield-related traits, such as KL or kernel number per row (KNPR), KW and 10-kernel thickness (KT) (Peng et al., 2011). In this study, we used an interactive usage model, FarmCPU instead of MLM or CMLM, in GWAS, which could exclude the false positive associations exactly. And the Q–Q plots also suggested that the false positive associations in this study were well controlled for the GWAS of the five traits across different environments. Then, we filtrated 20 unique loci (SNPs) at *P* < 3.95E-05 level among 97 SNPs that were associated with five ear-related traits. The twenty consistent SNPs about ear and kernel traits were found at the same chromosome intervals by integrating the previously reported QTLs information, among which four SNPs for KL were located at bin 3.07, 6.04, and 7.02 (2), three for KW at bin 6.07, 10.00, and 10.07, one for EL at bin 7.03, seven for ED at bin 1.10, 3.09, 4.05, 5.03, 8.03 (2), and 10.03, and five for CD at bin 1.05, 1.08, 1.10, 1.12, and 8.03, respectively. Especially, the SNP for KL at bin 7.02 was common in two environments. Besides, another SNP at bin 8.03 (PZE_108042082) was detected both for ED and CD, indicating that ED and CD may have same genetic basis, which was supported by the significant correlations of the two traits in our research materials. Therefore, our results would provide important information for further fine mapping yield-related genes, thus to reveal corresponding molecular mechanisms.

In addition, four functional genes detected are related to ubiquitin (Ub), which is widely existing in eukaryotes and highly conservative. The most important gene, GRMZM2G015287, encoded ubiquitin-conjugating enzyme E2N, and the other three separately encoded ubiquitin-activating enzyme E1 2, ubiquitin carboxyl-terminal hydrolase 13, and E3 ubiquitin protein ligase DRIP2. Ubiquitination pathway is an important regulatory process in plant biological activities including growth and development, response to biological and abiotic stress signals, etc. The covalent attachment of Ub to target proteins involves a three kinds of enzymes (ubiquitin-activating enzyme E1, ubiquitin-conjugating enzyme E2 and ubiquitin ligase E3) and a series of reactions via the transfer of thioester linkage between these enzymes (Ramadan et al., 2015). Previous studies have found that E3 is an important specific identification factor in regulating seed size during the process of ubiquitination (Hershko and Ciechanover, 1998). The gene *GW2* in rice could decrease cell division, associated with grain width, grain weight and growth period, codes a RING-type protein with E3 ubiquitin ligase activity that locating in the cytoplasm and degradating zymolytes by anchoring them into proteasomes and ultimately decreasing cell division.

The candidate genes mined in this study suggested that development of grain was related to ubiquitination pathway, then grain and related traits affect the maize yield. Jiang et al. (2015) found genes of *ZmGS3-CHR1-1, ZmGS3-CHR1-2, ZmGS3-CHR2, ZmGS3-CHR7, ZmGS3-CHR4*, and *ZmGS3-CHR5* that homologous with ring E3 ubiquitin-protein ligase gene *GS3* and *GS2* in rice by integrating meta-QTLs of ear row number and grain weight reported in major journals over 1994–2012. The gene *GW2* in rice could decrease cell division, and loss of *GW2* function would result in a larger and wider spikelet hull, which accelerated grain milk filling rate and enhanced grain width, weight and yield (Song et al., 2007; Yan S. et al., 2011). *WY3*, alleles of *GW2*, can significantly increase grain width and 1000-grain weight, resulting in the output raise of single plant. *D3*, a gene that encoding ubiquitin E3, was associated with ear number and plant height (Ishikawa et al., 2005) and encoded an F-box leucine-rich repeat (*LRR*) protein which can inhibit the activity of rice tiller buds, maintain their dormancy and participate in the dark-induced leaf senescence process and hydrogen-induced leaf cell death process (Yan H.F. et al., 2007; Falcon de Longevialle et al., 2008). So, four functional genes detected are related to ubiquitin, which is widely existing in eukaryotes and highly conservative.

Except that, two novel genes *PRFB1* (GRMZM2G398608) encoding peptide chain release factor PrfB1 in chloroplast (AtPrfB1) and GRMZM2G103843 encoding fructokinase-2 were found. *PRFB1* responses to the peptide chain termination codon UGA, which is necessary for the proper translation and stability of UGA-containing polycistronic transcripts in chloroplasts. So *AtPrfB1* participates in the biological processes of plastid organization (Meurer et al., 1996), RNA processing and translational termination (Meurer et al., 2002). Fructokinase-2 encoded by gene *FRK1* in maize, may play an important role in fructose metabolic process, thus maintaining the flux of carbon toward starch formation during seed development (Zhang et al., 2003; Riggs et al., 2017).

## Conclusion

Genetic diversity and GWAS of maize ear traits were performed in a panel of 292 inbred lines. And 20 significant SNPs associated with kernel size and grain yield were detected using FarmCPU software. Among them, a candidate genes on chromosome 1 was related to ubiquitin, and two novel candidate genes, (GRMZM2G398608 and GRMZM2G103843) related with ED and CD, were also explored in the study. Bioinformatics analysis showed that gene *PRFB1* (GRMZM2G398608) encode peptide chain release factor *PrfB1* in chloroplast (*AtPrfB1*) and GRMZM2G103843 encoded fructokinase-2. Besides, a MYB-CC type transfactor and a gene encoding phosphate starvation response protein 3 were found to be associated with KL and ED, respectively. These results would be helpful for understanding the relationship between yield and the ear-traits in maize.

## Author Contributions

X-YSo and M-AZ conceived and designed the study. M-AZ, X-MZ, X-YSh, Y-HP, X-MG, and JL performed the experiments, phenotyped the populations and field work. X-MZ and X-YSh contributed to genotype and phenotype data analysis. X-YSh conducted the meta-analysis. X-MZ drafted the manuscript. All authors read and approved the final manuscript.

## Conflict of Interest Statement

The authors declare that the research was conducted in the absence of any commercial or financial relationships that could be construed as a potential conflict of interest.

## Footnotes

1 https://brcwebportal.cos.ncsu.edu/powermarker/

2 http://www.maizegenetics.net/tassel

3 http://pritch.bsd.uchicago.edu/structure.html

4 http://www.maizegdb.org/

5 http://www.ncbi.nlm.nih.gov/gene

6 http://software.broadinstitute.org/software/igv/

7 http://www.broadinstitute.org/haploview

8 http://www.cnki.net/

## Supplementary Material

FIGURE S1 Manhattan plots and Quantile–Quantile (Q–Q) plots of **(A)** kernel length, **(B)** kernel width, **(C)** ear length, **(D)** ear diameter, **(E)** cob diameter in Qingzhou, 2015.

FIGURE S2 Manhattan plots and Quantile–Quantile (Q–Q) plots of **(A)** kernel length, **(B)** kernel width, **(C)** ear diameter, **(D)** cob diameter in Jiaozhou, 2016.

FIGURE S3 Manhattan plots and Quantile–Quantile (Q–Q) plots of **(A)** kernel length, **(B)** kernel width, **(C)** ear diameter, **(D)** cob diameter in Qingzhou, 2016.

TABLE S1 Phenotypic correlation coefficient estimates for each trait in Jiaozhou, 2016 (above diagonal) and Qingzhou, 2016 (below diagonal).

TABLE S2 List of 292 maize inbred lines.

TABLE S3 Complete imformation about SNPs and candidate genes using FarmCPU for GWAS.

## References

[B1] Bai H. G. (2014). *Methods of Kernel Traits Measurement and QTL Mapping and Analysis of Association.* Master’s thesis, Xinjiang Agricultural University, Urumchi.

[B2] Bai X. Zhao H. Huang Y. Xie W. Han Z. Zhang B. (2016). Genome-wide association analysis reveals different genetic control in panicle architecture between *indica* and *japonica* rice. *Plant Genome* 9 1–10. 10.3835/plantgenome2015.11.0115 27898816

[B3] Bommert P. Nagasawa N. S. Jackson D. (2013). Quantitative variation in maize kernel row number is controlled by the FASCIATED EAR2 locus. *Nat. Genet.* 45 334–337. 10.1038/ng.2534 23377180

[B4] Bournier M. Tissot N. Mari S. Boucherez J. Lacombe E. Briat J. F. (2013). *Arabidopsis* ferritin 1 (*AtFer1*) gene regulation by the phosphate starvation response 1 (AtPHR1) transcription factor reveals a direct molecular link between iron and phosphate homeostasis. *J. Biol. Chem.* 288 22670–22680. 10.1074/jbc.M113.482281 23788639 PMC3829352

[B5] Bradbury P. J. Zhang Z. Kroon D. E. Casstevens T. M. Ramdoss Y. Buckler E. S. (2007). TASSEL: software for association mapping of complex traits in diverse samples. *Bioinformatics* 23 2633–2635. 10.1093/bioinformatics/btm308 17586829

[B6] Chen D. H. Ronald P. (1999). A rapid DNA minipreparation method suitable for AFLP and other PCR applications. *Plant Mol. Biol. Rep.* 17 53–57. 10.1023/A:1007585532036

[B7] Chen G. F. Chen J. S. Tian J. C. (2015). Genome-Wide association analysis between SNP markers and plant height related traits in wheat. *Mol. Plant Breed.* 41 1500–1509. 10.3724/SP.J.1006.2015.01500

[B8] Cui Z. H. Luo J. H. Qi C. Y. Ruan Y. Y. Li J. Zhang A. (2016). Genome-wide association study (GWAS) reveals the genetic architecture of four husk traits in maize. *BMC Genomics* 17:946. 10.1186/s12864-016-3229-6 27871222 PMC5117540

[B9] Dai G. L. Cai Y. L. Xu D. L. Lv X. G. Wang G. Q. Wang J. G. (2009). QTL mapping for ear traits in maize (*Zea mays*, L.). *J. Southwest China Norm. Univ. Nat. Sci. Ed.* 34 133–138. 10.13718/j.cnki.xsxb.2009.05.030

[B10] Duvick D. N. (2005). Genetic progress in yield of United States Maize (*Zea mays* L.). *Maydica* 50 193–202.

[B11] Evanno G. Regnaut S. Goudet J. (2005). Detecting the number of clusters of individuals using the software STRUCTURE: a simulation study. *Mol. Ecol.* 14 2611–2620. 10.1111/j.1365-294X.2005.02553.x 15969739

[B12] Falcon de Longevialle A. Hendrickson L. Taylor N. Delannoy E. Lurin C. Badger M. (2008). The pentatricopeptide repeat gene OTP51 with two LAGLIDADG motifs is required for the cis-splicing of plastid ycf3 intron 2 in *Arabidopsis thaliana*. *Plant J.* 56 157–168. 10.1111/j.1365-313X.2008.03581.x 18557832

[B13] Goffinet B. Gerber S. (2000). Quantitative trait loci: a meta-analysis. *Genetics* 155 463–473. 10790417 10.1093/genetics/155.1.463 PMC1461053

[B14] Hershko A. Ciechanover A. (1998). The ubiquitin system. *Annu. Rev. Biochem.* 67 425–479. 10.1146/annurev.biochem.67.1.425 9759494

[B15] Hu L.-z. Liu J.-g. Guo J.-j. (2010). QTL analysis of ear traits based on BC2F2 population in maize (*Zea may* L.). *Acta Agric. Bar. Sin.* 25 73–77. 10.7668/hbnxb.2010.04.016

[B16] Ishikawa S. Maekawa M. Arite T. Onishi K. Takamure I. Kyozuka J. (2005). Suppression of tiller bud activity in tillering dwarf mutants of rice. *Plant Cell Physiol.* 46 79–86. 10.1093/pcp/pci022 15659436

[B17] Jiang L. Ge M. Zhao H. Zhang T. (2015). Analysis of heterosis and quantitative trait loci for kernel shape related traits using triple testcross population in maize. *PLoS One* 10:e0124779. 10.1371/journal.pone.0124779 25919458 PMC4412835

[B18] Kong M. L. Li C. Y. Sun Q. P. Lu M. Wang W. X. Pan J. B. (2014). Isolation and expression analysis of the E3 ubiquitin ligase encoding gene ZmGW2-1 in maize. *J. Anhui Agric. Univ.* 41 1004–1011. 10.13610/j.cnki.1672-352x.20141029.009 15316079

[B19] Li C. H. Li Y. X. Sun B. C. Peng B. Liu C. Liu Z. Z. (2013). Quantitative trait loci mapping for yield components and kernel-related traits in multiple connected RIL populations in maize. *Euphytica* 193 303–316. 10.1007/s10681-013-0901-7

[B20] Li C. Y. Kong M. L. Sun Q. P. Lu M. Wang W. X. Pan J. B. (2014). Cloning of homologous gene ZmGS5 in maize based on OsGS5. *J. Beijing Univ. Agric.* 29 14–17.

[B21] Li H. Peng Z. Y. Yang X. H. Wang W. D. Fu J. J. Wang J. H. (2012). Genome-wide association study dissects the genetic architecture of oil biosynthesis in maize kernels. *Nat. Genet.* 45 43–50. 10.1038/ng.2484 23242369

[B22] Li K. Wang H. Hu X. Liu Z. Wu Y. Huang C. (2016). Genome-wide association study reveals the genetic basis of stalk cell wall components in maize. *PLoS One* 11:e0158906. 10.1371/journal.pone.0158906 27479588 PMC4968789

[B23] Li X. Zhou Z. Ding J. Wu Y. Zhou B. Wang R. (2016). Combined linkage and association mapping reveals QTL and candidate genes for plant and ear height in maize. *Front. Plant Sci.* 7:833. 10.3389/fpls.2016.00833 27379126 PMC4908132

[B24] Li Y. X. Wang Y. Shi Y. S. Song Y. C. Wang T. Y. Li Y. (2009). Correlation analysis and QTL mapping for traits of kernel structure and yield components in maize. *Sci. Agric. Sin.* 42 408–418.

[B25] Li Y. Y. (2007). *The Genes Functional Compare Analysis between the OSPHR2 and ATPHRI.* Master’s thesis, Zhejiang University, Hangzhou.

[B26] Lipka A. E. Tian F. Wang Q. S. Peiffer J. Li M. Bradbury P. J. (2012). GAPIT: genome association and prediction integrated tool. *Bioinformatics* 28 2397–2399. 10.1093/bioinformatics/bts444 22796960

[B27] Li J. Z. Zhang Z. W. Li Y. L. Wang Q. L. Zhou Y. G. (2011). QTL consistency and meta-analysis for grain yield components in three generations in maize. *Theor. Appl. Genet.* 122 771–782. 10.1007/s00122-010-1485-4 21063866

[B28] Li Y. L. Niu S. Z. Dong Y. B. Cui D. Q. Wang Y. Z. Liu Y. Y. (2007). Identification of trait-improving quantitative trait loci for grain yield components from a dent corn inbred line in an advanced backcross BC_2_F_2_ population and comparison with its F_2_:_3_ population in popcorn. *Theor. Appl. Genet.* 115 129–140. 10.1007/s00122-007-0549-6 17492267

[B29] Liu C. L. Hao Z. F. Zhang D. G. Xie C. X. Li M. S. Zhao X. C. (2015). Genetic properties of 240 maize inbred lines and identity-by-descent segments revealed by high-density DNP markers. *Mol. Breed.* 35 146–157. 10.1007/s11032-015-0344-z

[B30] Liu K. Muse S. V. (2005). PowerMaker: an integrated analysis environment for genetic maker analysis. *Bioinformatics* 21 2128–2129. 10.1093/bioinformatics/bti282 15705655

[B31] Liu L. Du Y. F. Shen X. M. Li M. L. Sun W. Huang J. (2015). KRN4 controls quantitative variation in maize kernel row number. *PLoS Genet.* 11:e1005670. 10.1371/journal.pgen.1005670 26575831 PMC4648495

[B32] Liu N. Xue Y. D. Guo Z. Y. Li W. H. Tang J. H. (2016). Genome-wide association study identifies candidate genes for starch content regulation in maize kernels. *Front. Plant Sci.* 7:1046. 10.3389/fpls.2016.01046 27512395 PMC4961707

[B33] Liu X. H. He S. L. Zheng Z. P. Huang Y. B. Tan Z. B. Wu X. (2010). QTL identification for row number per ear and grain number per row in maize. *Maydica* 55 127–133.

[B34] Liu X. L. (2015). *Development of an Iterative Usage of Fixed Effect and Random Effect Models for Powerful and Efficient Genome-Wide Association Studies.* Master’s thesis, Huazhong agricultural University, Wuhan.

[B35] Liu Y. (2013). *QTL Mapping and Genetic Analysis of Kernel Size and Yield Components in Maize.* Master’s thesis, Huazhong Agricultural University, Wuhan.

[B36] Liu Y. Wang L. Sun C. Zhang Z. Zheng Y. Qiu F. (2014). Genetic analysis and major QTL detection for maize kernel size and weight in multi-environments. *Theor. Appl. Genet.* 127 1019–1037. 10.1007/s00122-014-2276-0 24553962

[B37] Lü X.-g. Cai Y.-l. Chen T.-q. Xu D.-l. Wang W.-l. Liu Z.-z. (2008). QTL mapping for ear traits in maize (*Zea mays* L.). *J. Southwest China Norm. Univ. (Nat. Sci. Edn.)* 34 133–138. 10.13718/j.cnki.xdzk.2008.02.021

[B38] Lu Y. L. Shah T. Hao Z. F. Taba S. Zhang S. H. Gao S. B. (2011). Comparative SNP and haplotype analysis reveals a higher genetic diversity and rapider LD decay in tropical than temperate germplasm in maize. *PLoS One* 6:e24861. 10.1371/journal.pone.0024861 21949770 PMC3174237

[B39] Ma X. S. Feng F. J. Wei H. B. Mei H. W. Xu K. Chen S. J. (2016). Genome-wide association study for plant height and grain yield in rice under contrasting moisture regimes. *Front. Plant Sci.* 7:1801. 10.3389/fpls.2016.01801 27965699 PMC5126757

[B40] Mammadov J. Sun X. Gao Y. Ochsenfeld C. Bakker E. Ren R. (2015). Combining powers of linkage and association mapping for precise dissection of QTL controlling resistance to gray leaf spot disease in maize (*Zea mays* L.). *BMC Genomics* 10:916. 10.1186/s12864-015-2171-3 26555731 PMC4641357

[B41] Mendesmoreira P. Alves M. L. Satovic Z. Dos Santos J. P. Santos J. N. Souza J. C. (2015). Genetic architecture of ear fasciation in maize (*Zea mays* L.) under QTL scrutiny. *PLoS One* 10:e0124543. 10.1371/journal.pone.0124543 25923975 PMC4414412

[B42] Messmer R. Fracheboud Y. Banziger M. Vargas M. Stamp P. Ribaut J. M. (2009). Drought stress and tropical maize: QTL-by-environment interactions and stability of QTLs across environments for yield components and secondary traits. *Theor. Appl. Genet.* 119 913–930. 10.1007/s00122-009-1099-x 19597726

[B43] Meurer J. Berger A. Westhoff P. (1996). A nuclear mutant of Arabidopsis with impaired stability on distinct transcripts of the plastid psbB, psbD/C, ndhH, and ndhC operons. *Plant Cell* 8 1193–1207. 10.1105/tpc.8.7.1193 8768377 PMC161203

[B44] Meurer J. Lezhneva L. Amann K. Gödel M. Bezhani S. Sherameti I. (2002). A peptide chain release factor 2 affects the stability of UGA-containing transcripts in Arabidopsis chloroplasts. *Plant Cell* 14 3255–3269. 10.1105/tpc.006809 12468741 PMC151216

[B45] Moseley J. L. Chang C. W. Grossman A. R. (2006). Genome-based approaches to understanding phosphorus deprivation responses and PSR1 control in *Chlamydomonas reinhardtii*. *Eukaryot. Cell* 5 26–44. 10.1128/EC.5.1.26-44.2006 16400166 PMC1360252

[B46] Okuda K. Myouga F. Motohashi R. Shinozaki K. Shikanai T. (2007). Conserved domain structure of pentatricopeptide repeat proteins involved in chloroplast RNA editing. *Proc. Natl. Acad. Sci. U.S.A.* 104 8178–8183. 10.1073/pnas.0700865104 17483454 PMC1876591

[B47] Peng B. Li Y. Wang Y. Liu C. Liu Z. Tan W. (2011). QTL analysis for yield components and kernel-related traits in maize across multi-environments. *Theor. Appl. Genet.* 122 1305–1320. 10.1007/s00122-011-1532-9 21286680

[B48] Pritchard J. K. Stephens M. Donnelly P. (2000). Inference of population structure using multilocus genotype data. *Genetics* 155 945–959. 10835412 10.1093/genetics/155.2.945 PMC1461096

[B49] Qi Z. M. Sun Y. N. Wang J. L. Zhang D. W. Liu C. Y. Hu G. H. (2011). Meta-Analysis of 100-seed weight QTLs in soybean. *Agric. Sci. China* 10 327–334. 10.1016/S1671-2927(11)60011-4 22740134

[B50] Qin W. W. Li Y. X. Li C. H. Chen L. Wu X. Bai N. (2015). QTL mapping for kernel related traits based on a high-density genetic map. *J. Crops* 9 1510–1518. 10.3724/SP.J.1006.2015

[B51] Raihan M. S. Liu J. Huang J. Guo H. Pan Q. Yan J. (2016). Multi-environment QTL analysis of grain morphology traits and fine mapping of a kernel-width QTL in Zheng58 × SK maize population. *Theor. Appl. Genet.* 129 1465–1477. 10.1007/s00122-016-2717-z 27154588

[B52] Ramadan A. Nemoto K. Seki M. Shinozaki K. Takeda H. Takahashi H. (2015). Wheat germ-based protein libraries for the functional characterisation of the Arabidopsis E2 ubiquitin conjugating enzymes and the RING-type E3 ubiquitin ligase enzymes. *BMC Plant Biol.* 15:275. 10.1186/s12870-015-0660-9 26556605 PMC4641371

[B53] Riggs J. W. Cavales P. C. Chapiro S. M. Calli S. J. (2017). Identification and biochemical characterization of the fructokinase gene family in *Arabidopsis thaliana*. *BMC Plant Biol.* 17:83. 10.1186/s12870-017-1031-5 28441933 PMC5405513

[B54] Rogers J. S. (1972). Measures of genetic similarity and genetic distance. *Stud. Genet. Univ. Texas Publ.* 7213 145–153.

[B55] Schnable P. S. Ware D. Fulton R. S. Stein J. C. Wei F. Pasternak S. (2009). The B73 maize genome:- complexity, diversity, and dynamics. *Science* 326 1112–1115. 10.1126/science.1178534 19965430

[B56] Song X. J. Huang W. Shi M. Zhu M. Z. Lin H. X. (2007). A QTL for rice grain width and weight encodes a previously unknown RING-type E3 ubiquitin ligase. *Nat. Genet.* 39 623–630. 10.1038/ng2014 17417637

[B57] Tian Y. H. Zhang Y. L. Fan Z. X. Tao Y. S. (2014). Bioinformatics analysis of orthologous maize gene based on rice kernel length gene OsPPKL1. *Guizhou Agric. Sci.* 42 1–6.

[B58] Wang H. Li K. Hu X. Liu Z. Wu Y. Huang C. (2016). Genome-wide association analysis of forage quality in maize mature stalk. *BMC Plant Biol.* 16:227. 10.1186/s12870-016-0919-9 27769176 PMC5073832

[B59] Wang L. W. (2015). *Fine Mapping of the Main Effect QTL qKL9 of Kernel Length.* Master’s thesis, Huazhong Agricultural University, Wuhan.

[B60] Wang Q. X. Xie W. B. Xing K. J. Yan J. Meng X. J. Li X. L. (2015). Genetic architecture of natural variation in rice chlorophyll content revealed by a genome-wide association study. *Mol. Plant* 8 946–957. 10.1016/j.molp.2015.02.014 25747843

[B61] Wang X. Wang H. Liu S. Ferjani A. Li J. Yan J. (2016). Genetic variation in ZmVPP1 contributes to drought tolerance in maize seedlings. *Nat. Genet.* 48 1233–1241. 10.1038/ng.3636 27526320

[B62] Wu X. Li Y. Shi Y. Song Y. Wang T. Huang Y. (2014). Fine genetic characterization of elite maize germplasm using high-throughput SNP genotyping. *Theor. Appl. Genet.* 127 621–631. 10.1007/s00122-013-2246-y 24343198

[B63] Xia X. Ma Y. C. Bai Q. H. Feng Y. Y. Wang S. Y. Wang X. (2016). Cloning and expression analysis of ZmMADS-RIN gene for regulating the kernel development of maize. *Acta Agron. Sin.* 42 1656–1665. 10.3724/SP.J.1006.2016.01656 16478053

[B64] Xiao Y. Tong H. Yang X. Xu S. Pan Q. Qiao F. (2016). Genome-wide dissection of the maize ear genetic architecture using multiple populations. *New Phytol.* 210 1095–1106. 10.1111/nph.13814 26715032

[B65] Yan H. F. Saika H. Maekawa M. Takamure I. Tsutsumi N. Kyozuka J. (2007). Rice tillering dwarf mutant dwarf3 has increased leaf longevity during darkness-induced senescence or hydrogen peroxide-induced cell death. *Genes Genet. Syst.* 82 361–366. 10.1266/ggs.82.361 17895586

[B66] Yan J. B. Warburton M. Crouch J. (2011). Association mapping for enhancing maize (*Zea mays* L.) genetic improvement. *Crop Sci.* 51 433–449. 10.2135/cropsci2010.04.0233

[B67] Yan S. Zou G. Li S. Wang H. Liu H. Zhai G. (2011). Seed size is determined by the combinations of the genes controlling different seed characteristics in rice. *Theor. Appl. Genet.* 123 1173–1181. 10.1007/s00122-011-1657-x 21805338

[B68] Yang C. Zhang L. Jia A. Rong T. (2016). Identification of QTL for maize grain yield and kernel-related traits. *J. Genet.* 95 239–247. 10.1007/s12041-016-0628-z 27350665

[B69] Yang X. J. (2008). *Mapping of Quantitative Trait Loci (QTL) and Genetic Effect for Important Traits with an Elite Maize Hybrid.* Master’s thesis, Xinjiang Agricultural University, Urumchi.

[B70] Zhang H. X. Weng J. F. Zhang X. C. Liu C. L. Yong H. J. Hao Z. F. (2014). Genome-wide association analysis of kernel row number in maize. *Acta Agron. Sin.* 40:1 10.3724/SP.J.1006.2014.00001

[B71] Zhang J. H. Liu Z. Z. Zhu Y. L. (2009). QTL mapping for ear traits under different densities using DH population of maize. *J. Hebei Agric. Univ.* 32 1–6.

[B72] Zhang S. R. Nichols S. E. Dong J. G. (2003). Cloning and characterization of two fructokinases from maize. *Plant Sci.* 165 1051–1058. 10.1016/0021-9150(95)05574-G

[B73] Zhang X. Zhang H. Li L. J. Lan H. Ren Z. Y. Liu D. (2016). Characterizing the population structure and genetic diversity of maize breeding germplasm in Southwest China using genome-wide SNP markers. *BMC Genomics* 17:697. 10.1186/s12864-016-3041-3 27581193 PMC5007717

