# Supplementary figures and images for: Genetic Diversity and Genome-Wide Association Study of Major Ear Quantitative Traits Using High-Density SNPs in Maize
Source: Front Plant Sci. 2018 Jul 9;9:966. doi: 10.3389/fpls.2018.00966 (PMC6046616; doi:10.3389/fpls.2018.00966)

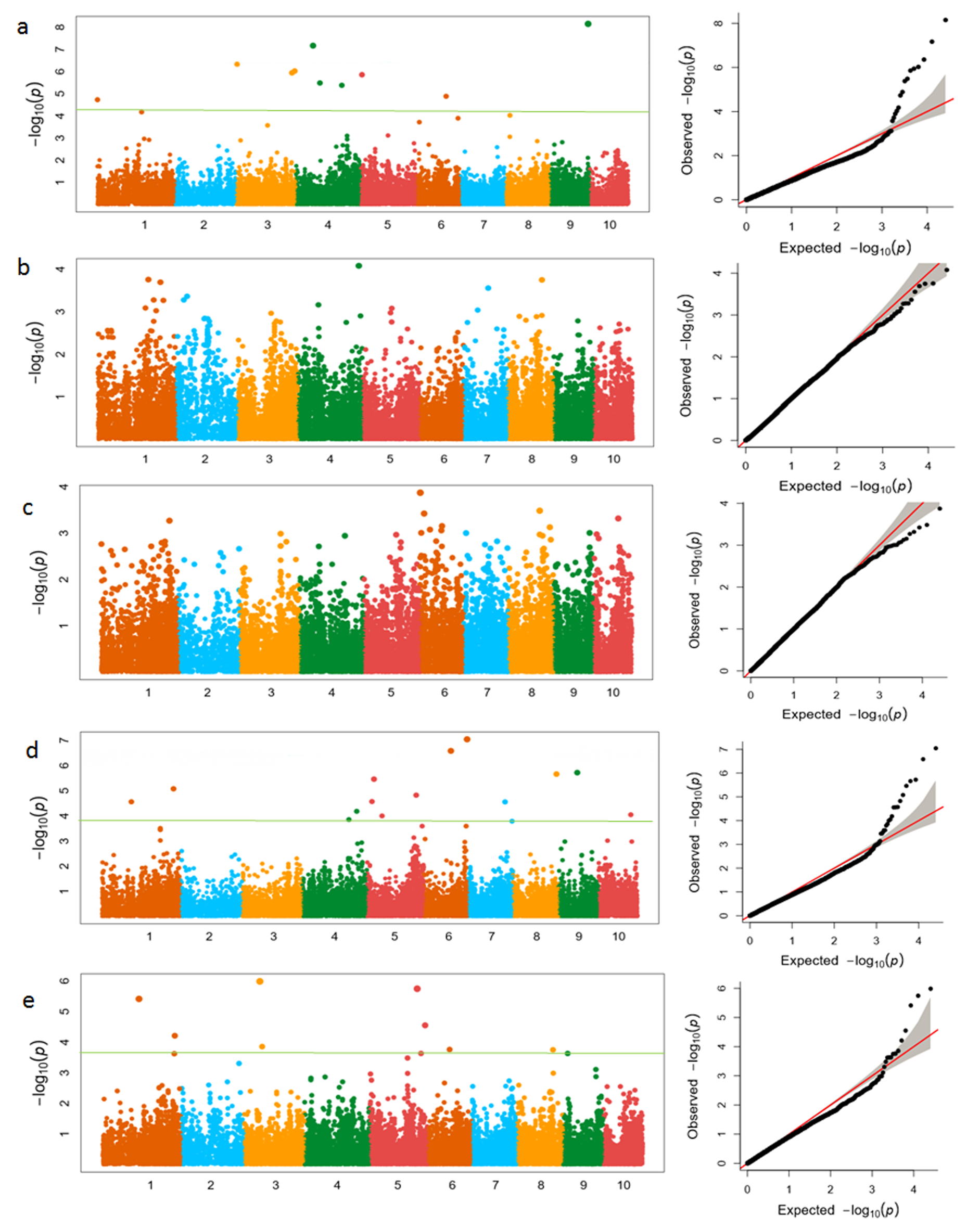

Supplement: FIGURE S1 — Manhattan plots and Quantile–Quantile (Q–Q) plots of (A) kernel length, (B) kernel width, (C) ear length, (D) ear diameter, (E) cob diameter in Qingzhou, 2015. [file Image_1.TIF]

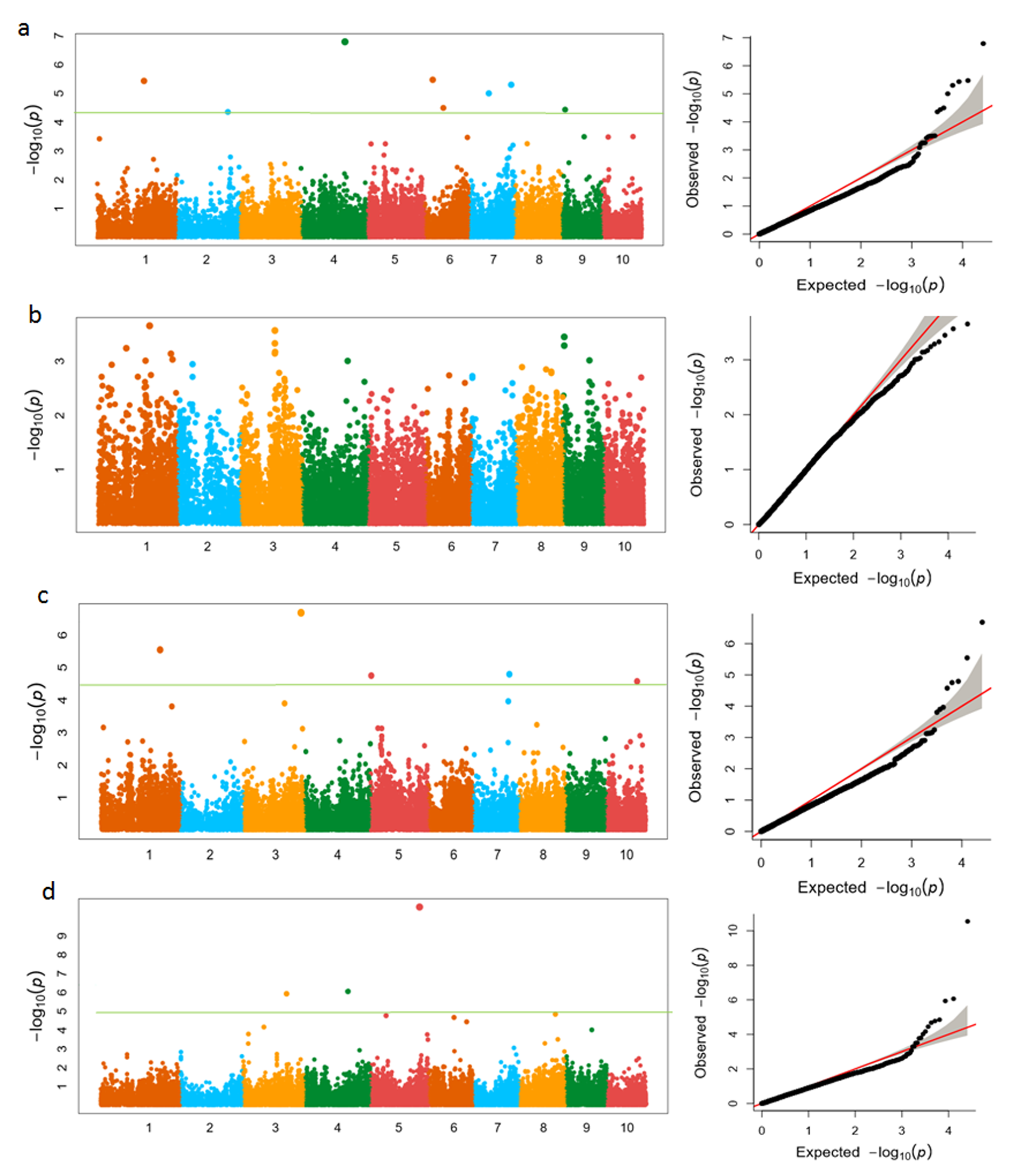

Supplement: FIGURE S2 — Manhattan plots and Quantile–Quantile (Q–Q) plots of (A) kernel length, (B) kernel width, (C) ear diameter, (D) cob diameter in Jiaozhou, 2016. [file Image_2.TIF]

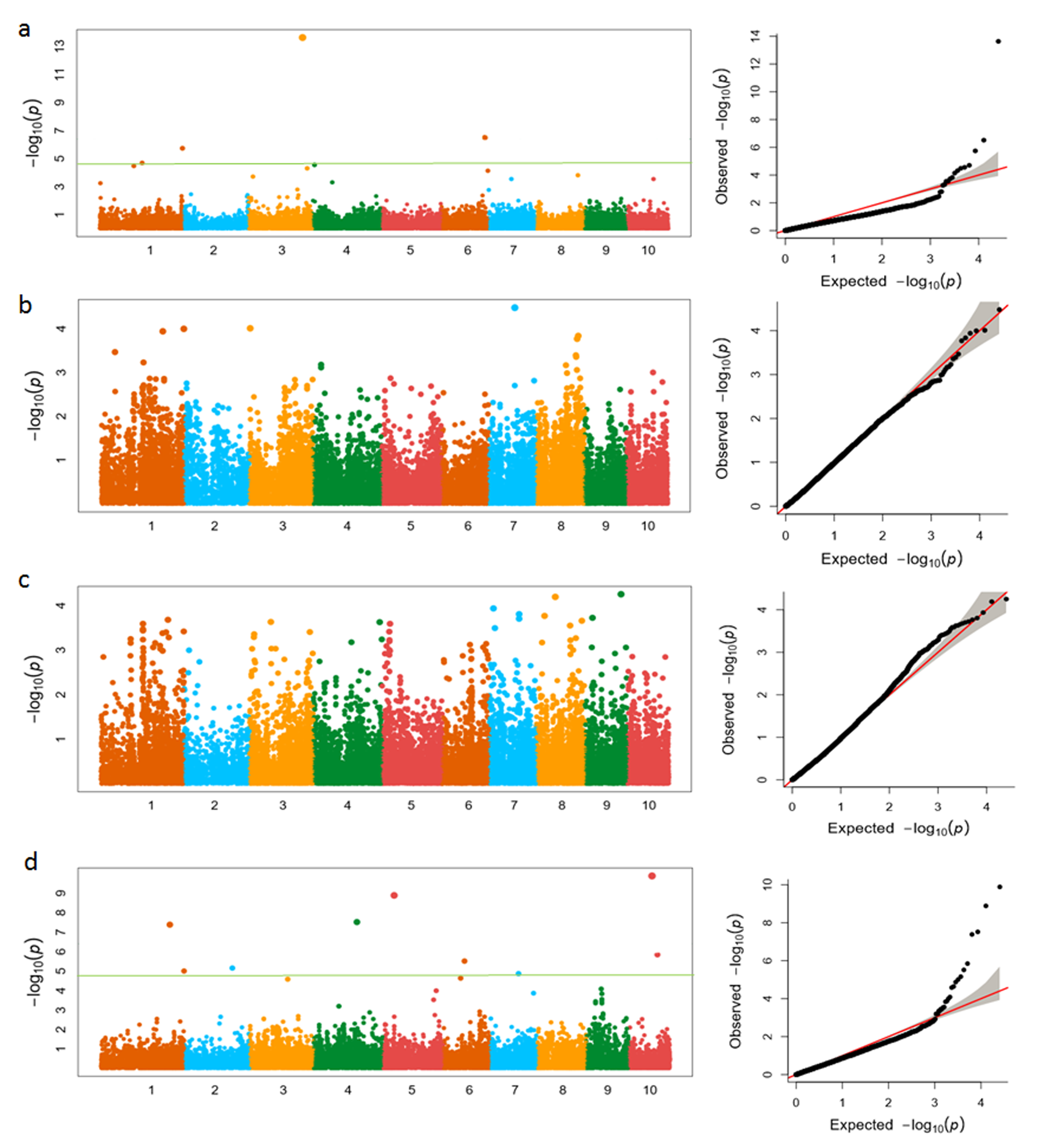

Supplement: FIGURE S3 — Manhattan plots and Quantile–Quantile (Q–Q) plots of (A) kernel length, (B) kernel width, (C) ear diameter, (D) cob diameter in Qingzhou, 2016. [file Image_3.TIF]
