# Supplementary material for: Genetic Diversity and Genome-Wide Association Study of Major Ear Quantitative Traits Using High-Density SNPs in Maize
Source: Front Plant Sci. 2018 Jul 9;9:966. doi: 10.3389/fpls.2018.00966 (PMC6046616; doi:10.3389/fpls.2018.00966)
Supplement: TABLE S1 — Phenotypic correlation coefficient estimates for each trait in Jiaozhou, 2016 (above diagonal) and Qingzhou, 2016 (below diagonal). [file Table_1.DOCX]

**TABLE S1** | Phenotypic correlation coefficient estimates for each trait in Jiaozhou, 2016 (above diagonal) and Qingzhou, 2016 (below diagonal).

| Trait | KL | KW | ED | CD |
| --- | --- | --- | --- | --- |
| KL |  | 0.227** | 0.552** | 0.081^ns^ |
| KW | 0.260** |  | 0.211** | 0.213** |
| ED | 0.652** | 0.316** |  | 0.654** |
| CD | 0.154** | 0.344** | 0.715** |  |

*KL, kernel length; KW, kernel width; EL, ear length; ED, ear diameter; CD, cob diameter.*

*** , significant at p<0.01, *, significant at p<0.05; ns, not significant.*
