# Supplementary material for: Genetic Diversity and Genome-Wide Association Study of Major Ear Quantitative Traits Using High-Density SNPs in Maize
Source: Front Plant Sci. 2018 Jul 9;9:966. doi: 10.3389/fpls.2018.00966 (PMC6046616; doi:10.3389/fpls.2018.00966)
Supplement: TABLE S2 — List of 292 maize inbred lines. [file Table_2.DOCX]

**TABLE S2** | List of 292 maize inbred lines.

| Number | Name | Number | Name | Number | Name | Number | Name |
| --- | --- | --- | --- | --- | --- | --- | --- |
| 1 | Ye478 | 74 | LY023 | 147 | L219 | 220 | Qi205 |
| 2 | Zao49 | 75 | LY024 | 148 | L502-196 | 221 | Qi318 |
| 3 | E28 | 76 | LY025 | 149 | LD3162 | 222 | 107X |
| 4 | B73 | 77 | LY026 | 150 | LX9801 | 223 | 11N597 |
| 5 | 444 | 78 | LY028 | 151 | M01 | 224 | 129-2405 |
| 6 | P138 | 79 | LY029 | 152 | M4 | 225 | ReBS11 |
| 7 | P170 | 80 | LY030 | 153 | MJ02 | 226 | Renbai |
| 8 | 853 | 81 | LY031 | 154 | ML-1 | 227 | Santuan |
| 9 | 7922 | 82 | LY032 | 155 | zm5536 | 228 | Shannong206a |
| 10 | 81162 | 83 | LY033 | 156 | zm5537 | 229 | Shannong206b |
| 11 | K12 | 84 | LY034 | 157 | zm5538 | 230 | Shan814 |
| 12 | Hainan1/6 | 85 | LY035 | 158 | zm5539 | 231 | Shen137 |
| 13 | Ji846 | 86 | LY036 | 159 | zm5540 | 232 | Shendan16F |
| 14 | Qi319 | 87 | LY037 | 160 | zm5541 | 233 | Shengyu88m |
| 15 | Qingnong105F | 88 | LY038 | 161 | zm5542 | 234 | Shunyao7hao |
| 16 | Qingnong105M | 89 | LY039 | 162 | zm5543 | 235 | Sun1 |
| 17 | JingD24 | 90 | LY042 | 163 | Ao20-3 | 236 | Sun2 |
| 18 | Liangyu88m | 91 | LY044 | 164 | o894-2 | 237 | Sun3 |
| 19 | 13tian-3 | 92 | LY045 | 165 | Bai515 | 238 | Tian06-261 |
| 20 | 319B | 93 | LY046 | 166 | Bai515 | 239 | Tian16 |
| 21 | 335Xuan | 94 | LY047 | 167 | Bao-1 | 240 | Tian-2 |
| 22 | 340G | 95 | LY049 | 168 | Benyu15 | 241 | Zhong102 |
| 23 | 414Xi | 96 | LY050 | 169 | Chang7-2G | 242 | Zhongdan909X |
| 24 | 496WP | 97 | LY054 | 170 | Chao6X | 243 | Zhongxi091 |
| 25 | 78599Xuan | 98 | K12HF304 | 171 | Zhaobai-1 | 244 | Zhunuo-7 |
| 26 | LY055 | 99 | K12HF76 | 172 | Chong17-2 | 245 | Zyao515 |
| 27 | LY056 | 100 | K36 | 173 | Chong17-2 | 246 | 92Huang40 |
| 28 | LY057 | 101 | K6H4057 | 174 | Dan340 | 247 | A632 |
| 29 | 87-20 | 102 | K6H6079 | 175 | Dan638 | 248 | AMD43X |
| 30 | K12-452 | 103 | K6H6179 | 176 | Dan638 | 249 | FeiLB-2 |
| 31 | K12-512 | 104 | K6H6784 | 177 | Dan998 | 250 | Nonghua101m |
| 32 | K12-526 | 105 | K6H9103 | 178 | Dansy 3-1 | 251 | Nnuo-2 |
| 33 | K12-76 | 106 | K8112 | 179 | Danhuang25 | 252 | Nuo-3 |
| 34 | K12HF184 | 107 | KN-1 | 180 | Du6607 | 253 | Nuo-4 |
| 35 | K910G | 108 | KN-1m | 181 | FeiLB-1 | 254 | Nuo-5 |
| 36 | KHL88 | 109 | LY059 | 182 | WYH-2 | 255 | Pengtian11-A |
| 37 | CML13 | 110 | LY060 | 183 | WYH-3 | 256 | Xi1-4 |
| 38 | CML84 | 111 | LY061 | 184 | X178 | 257 | Xia514 |
| **TABLE S2** \| Continued   \| Number \| Name \| Number \| Name \| Number \| Name \| Number \| Name \| \| --- \| --- \| --- \| --- \| --- \| --- \| --- \| --- \| | | | | | | | |
| 39 | CML99 | 112 | LY062 | 185 | XD28 | 258 | Xia844 |
| 40 | CML199 | 113 | LY064 | 186 | Y53 | 259 | Xia987 |
| 41 | CML255 | 114 | LY065 | 187 | YM-8 | 260 | Xia996 |
| 42 | CML299 | 115 | LY066 | 188 | YWH67 | 261 | Xian96 |
| 43 | CML306 | 116 | LY068 | 189 | zm5535 | 262 | XianfengXuan |
| 44 | CML385 | 117 | LY069 | 190 | FeiLB-3 | 263 | Xianyu698X |
| 45 | D811 | 118 | LY070 | 191 | Feng273 | 264 | Xin1391 |
| 46 | DH7823 | 119 | LY071 | 192 | Fu96 | 265 | XinDH |
| 47 | Ex | 120 | LY073 | 193 | HaiY18 | 266 | Yan103 |
| 48 | LY001 | 121 | LY074 | 194 | Hainan-2 | 267 | Yan172 |
| 49 | LY002 | 122 | LY11-11 | 195 | Hua160 | 268 | 254 |
| 50 | LY006 | 123 | LYM1 | 196 | HuayuW13 | 269 | Yi67 |
| 51 | LY007 | 124 | LYM2 | 197 | Huang5 | 270 | Zao10 |
| 52 | LY009 | 125 | LYM3 | 198 | Huang515 | 271 | Zao10 |
| 53 | LY010 | 126 | LYM4 | 199 | Jichu1 | 272 | Zhao835 |
| 54 | LY013 | 127 | K_12_-146 | 200 | 107-8 | 273 | Zheng0510 |
| 55 | LY014 | 128 | K_12_-148 | 201 | jiM67 | 274 | Zheng58G |
| 56 | LY016 | 129 | K_12_-160 | 202 | Jizao48 | 275 | H231 |
| 57 | LY017 | 130 | K1_2_-176 | 203 | Jizaobai | 276 | H90 |
| 58 | LY018 | 131 | K_12_-272 | 204 | Jinhai5 | 277 | 189 |
| 59 | LY019 | 132 | K_12_-386 | 205 | 13H-342 | 278 | BC2433 |
| 60 | LY020 | 133 | HO-3-4 | 206 | 13H-375 | 279 | BM |
| 61 | LY021 | 134 | NF358 | 207 | Liao3162 | 280 | 230 |
| 62 | JH271 | 135 | NLEBM-4 | 208 | Liao3180 | 281 | D729 |
| 63 | K12B | 136 | OA1207 | 209 | liaoyu20mu | 282 | 244 |
| 64 | H901 | 137 | P1211-6 | 210 | Lu65 | 283 | FC521 |
| 65 | ML-3 | 138 | 488 | 211 | Mei24242 | 284 | FR218 |
| 66 | MLBJ | 139 | 785 | 212 | Mei338 | 285 | ML-2 |
| 67 | MQ-3 | 140 | S122 | 213 | Meikang-1 | 286 | Tie9010 |
| 68 | MY-4 | 141 | T123 | 214 | Meikang-2 | 287 | Tie98042 |
| 69 | MY6-8 | 142 | T29803 | 215 | Meikang-3 | 288 | TieX98042 |
| 70 | NF-35 | 143 | W42-2 | 216 | Meikang-4 | 289 | Tiedan9010 |
| 71 | TW263 | 144 | WL | 217 | Meixuan | 290 | Weihaibai |
| 72 | TW623 | 145 | L01125 | 218 | Tian-4 | 291 | Wei1122 |
| 73 | LY022 | 146 | L0167 | 219 | Pengtian33-A | 292 | Xixingnuo-6 |
