# Supplementary material for: Genetic Diversity and Genome-Wide Association Study of Major Ear Quantitative Traits Using High-Density SNPs in Maize
Source: Front Plant Sci. 2018 Jul 9;9:966. doi: 10.3389/fpls.2018.00966 (PMC6046616; doi:10.3389/fpls.2018.00966)
Supplement: TABLE S3 — Complete imformation about SNPs and candidate genes using FarmCPU for GWAS. [file Table_3.DOCX]

**TABLE S3** | Complete imformation about SNPs and candidate genes using FarmCPU for GWAS.

| Traits | Env | SNP ID | Bin | Position | P-value | Gene ID | Encoding |
| --- | --- | --- | --- | --- | --- | --- | --- |
| KL | LY15 | PZE_106099248 | 6.05 | 152,896,893 | 5.52E-09 | GRMZM2G067198 | Hypothetical protein |
|  |  | SYN34114 | 1.10 | 274,384,205 | 4.92E-07 | pco090181a | Transmembrane emp24 domain-containing protein p24beta3 |
|  |  | SYN39240 | 1.07 | 216,624,392 | 1.29E-05 |  |  |
|  |  | SYN9381 | 1.01 | 3,337,882 | 1.91E-05 | GRMZM2G426802 | Signal transducer |
|  |  | PZE_107042407 | 7.02 | 72,337,865 | 2.23E-05 | GRMZM2G173943 | MYB-CC type transfactor |
|  |  | PZE_103157525 | 3.08 | 208,046,069 | 3.28E-05 |  |  |
|  |  | PZE_108103787 | 8.06 | 158,021,459 | 3.94E-05 | LOC103636305 | Calcium-transporting ATPase 3, plasma Membrane-type-like |
|  |  | PZE_101167577 | 1.07 | 210,397,316 | 6.92E-05 | GRMZM2G389567 | Hypothetical protein |
|  | QZ15 | PZE_104042141 | 4.05 | 65,373,561 | 6.77E-08 |  |  |
|  |  | SYN15340 | 3.04 | 3,779,020 | 4.39E-07 |  |  |
|  |  | SYN35152 | 3.09 | 225,190,422 | 9.45E-07 | GRMZM2G071154 | DCD (Development and Cell Death) domain protein |
|  |  | PZE_103167491 | 3.08 | 214,728,947 | 1.14E-06 | pco110172(456) | Dihydroflavonol-4-reductase |
|  |  | SYNGENTA10208 | 5.01 | 6,944,755 | 1.39E-06 | GRMZM2G553379 | M15 protein |
|  |  | PZE_104053095 | 4.05 | 91,836,417 | 3.26E-06 |  |  |
|  |  | SYN2326 | 4.07 | 176,210,583 | 4.15E-06 | GRF3 | Growth-regulating factor 3-like |
|  |  | PZE_106062790 | 6.04 | 114,026,921 | 1.30E-05 | pco134695(574) | Protease Do-like 9 |
|  |  | SYN8296 | 1.00 | 988,243 | 1.86E-05 |  |  |
|  |  | PZE_101132519 | 1.05 | 170,773,078 | 6.64E-05 |  |  |
|  |  | PZE_108021008 | 8.02 | 20,006,404 | 9.41E-05 |  |  |
|  | JZ16 | PZE_104090186 | 4.06 | 165,069,099 | 1.62E-07 |  |  |
|  |  | SYN11572 | 1.06 | 175,911,365 | 3.68E-06 |  |  |
|  |  | PZE_107113353 | 7.04 | 156,454,515 | 4.99E-06 | GRMZM2G057260 | Transcription factor bHLH54 |
|  |  | PZE_107042407 | 7.02 | 72,337,865 | 9.93E-06 | GRMZM2G173943 | MYB-CC type transfactor |
|  |  | PZE_106030596 | 6.01 | 70,819,632 | 3.18E-05 | GRMZM2G364349 | Putative pectate lyase 3 |
|  |  | SYN38530 | 9.02 | 13,906,759 | 3.65E-05 | LOC100502328 | DBP transcription factor |
|  |  | SYN21432 | 2.07 | 190,585,974 | 4.38E-05 |  |  |
|  | QZ16 | PZE_103141524 | 3.07 | 195,218,947 | 2.31E-14 |  |  |
|  |  | PZE_106103773 | 6.06 | 155,376,702 | 3.06E-07 |  |  |
|  |  | PZE_101122743 | 1.05 | 153,374,195 | 1.99E-05 |  |  |
|  |  | PZE_104010504 | 4.02 | 7,700,318 | 2.78E-05 |  |  |
|  |  | PZE_101112762 | 1.05 | 123,552,755 | 3.28E-05 | GRMZM2G451856 | Mediator of RNA polymerase II transcription subunit 34 |
|  |  | PZE_103162737 | 3.08 | 211,743,217 | 4.85E-05 | GRMZM2G033515 | Seed maturation protein PM41 |
|  |  | SYN24811 | 6.07 | 167,147,049 | 7.22E-05 |  |  |
| KW | LY15 | PZE_110105598 | 9.06 | 146,673,417 | 7.38E-10 | GRMZM2G124502 | SWIB complex BAF60b domain-containing protein |
|  |  | SYN4309 | 6.07 | 165,411,234 | 6.09E-09 | GRMZM2G092475 | probable sodium/metabolite cotransporter BASS4, chloroplastic |
|  |  | PZE_110000228 | 9.00 | 642,928 | 1.54E-08 | GRMZM2G057441 | Ubiquitin-activating enzyme E1 2 |
|  |  | PZE_107037159 | 7.02 | 58,807,506 | 3.99E-07 |  |  |
|  |  | PZE_101145493 | 1.06 | 188,037,816 | 8.62E-07 | GRMZM2G161004 | Nucleic acid binding protein |
|  |  | PZE_103026868 | 3.04 | 19,507,611 | 2.83E-06 |  |  |
|  |  | SYN10351 | 1.10 | 281,492,863 | 6.25E-06 |  |  |
|  |  | PZE_108096610 | 8.06 | 151,407,794 | 1.97E-05 | GRMZM2G316907 | Probable LRR receptor-like serine/threonine-protein kinase At3g47570 |
|  |  | PZE_109059124 | 9.03 | 97,781,598 | 2.65E-05 |  |  |
|  |  | PZE_103078999 | 3.05 | 126,965,769 | 2.95E-05 |  |  |
|  | QZ15 | PZE_104139559 | 4.09 | 232,812,308 | 8.35E-05 | GRMZM2G029058 | Polyadenylate-binding protein-interacting protein 9 |
|  | QZ16 | PZE_107050623 | 7.02 | 94,659,009 | 3.32E-05 |  |  |
|  |  | SYN6756 | 3.01 | 3,655,418 | 9.82E-05 |  |  |
| EL | LY15 | PZE_107081628 | 7.03 | 136607023 | 6.61E-07 | GRMZM2G074386 | proteasome subunit beta type-1 |
| ED | LY15 | PZE_108042082 | 8.03 | 67245292 | 3.75E-09 | GRMZM2G015287 | Ubiquitin-conjugating enzyme E2 N |
|  |  | PZE_104069344 | 4.05 | 137099594 | 1.61E-08 | GRMZM2G179810 | Adenine phosphoribosyltransferase 2 |
|  |  | PZE_101048890 | 1.03 | 33,677,004 | 5.66E-08 |  |  |
|  |  | PZE_106015303 | 6.01 | 36,836,724 | 3.44E-07 |  |  |
|  |  | PZE_110044605 | 10.03 | 85,027,699 | 1.43E-06 | GRMZM2G126007 | UPF0481 protein At3g47200 |
|  |  | PZE_104124003 | 4.09 | 206956947 | 4.67E-06 | LOC100381748 | Ubiquitin carboxyl-terminal hydrolase 13 |
|  |  | PZE_106056547 | 6.04 | 110,465,686 | 4.85E-06 |  |  |
|  |  | PZE_108035132 | 8.03 | 49,110,637 | 5.68E-06 |  |  |
|  |  | PZE_101208406 | 1.09 | 258,034,687 | 1.17E-05 | GRMZM2G319281 | Acidic leucine-rich nuclear phosphoprotein 32 family |
|  |  | PZE_105041346 | 5.03 | 27,663,265 | 2.86E-05 |  |  |
|  | QZ15 | SYN4311 | 6.07 | 165,447,568 | 9.04E-08 |  |  |
|  |  | PZE_106052904 | 6.03 | 103,570,219 | 2.61E-07 | GRMZM2G310144 | ABC transporter A family member 7 |
|  |  | PZE_109044289 | 9.03 | 76,109,248 | 1.90E-06 | LOC103640029 | V-type proton ATPase subunit a1 |
|  |  | PZE_108119984 | 8.07 | 167,554,776 | 2.17E-06 |  |  |
|  |  | PZE_105040885 | 5.03 | 26,890,327 | 3.46E-06 |  |  |
|  |  | PZE_101223864 | 1.10 | 275,012,730 | 8.37E-06 | GRMZM2G018798 | E3 ubiquitin protein ligase DRIP2 |
|  |  | SYNGENTA6857 | 5.05 | 188,509,313 | 1.50E-05 | GRMZM2G178595 | E2F-associated phosphoprotein |
|  |  | SYN32845 | 5.03 | 19,076,912 | 2.68E-05 | GRMZM2G375984 | Protein DEK |
|  |  | PZE_101108171 | 1.05 | 114,182,209 | 2.74E-05 |  |  |
|  |  | SYNGENTA11834 | 7.03 | 146,534,090 | 2.77E-05 | GRMZM2G032003 | UDP-glucose pyrophosphorylase2 |
|  |  | SYN28068 | 4.08 | 202,250,929 | 6.55E-05 |  |  |
|  |  | PZE_110067406 | 10.04 | 123,976,464 | 8.94E-05 | GRMZM2G301246 | Poly(A)-specific ribonuclease PARN |
|  |  | PZE_105059330 | 5.03 | 58,165,601 | 9.97E-05 | LOC103626417 | Protein PHOSPHATE STARVATION RESPONSE 3 |
|  | JZ16 | PZE_103171163 | 3.09 | 218,272,659 | 2.07E-07 | GRMZM2G156158 | P-loop containing nucleoside triphosphate hydrolase |
|  |  | SYN16352 | 1.07 | 223,385,856 | 2.85E-06 | GRMZM2G345725 | Sphingoid long-chain bases kinase 1 |
|  |  | PZE_107084584 | 7.03 | 140,288,830 | 1.60E-05 |  |  |
|  |  | SYN15172 | 5.00 | 2,745,843 | 1.76E-05 | GRMZM2G165209 | Protein FLC EXPRESSOR |
|  |  | PZE_110061773 | 10.04 | 116,913,027 | 2.64E-05 | GRMZM2G054632 | Putative magnesium transporter MRS2-D |
| CD | LY15 | PZE_101255159 | 1.11 | 297,703,437 | 4.31E-08 | GRMZM2G084176 | Putative pentatricopeptide repeat-containing protein |
|  |  | PZE_108042082 | 8.03 | 67,245,292 | 6.64E-06 | GRMZM2G015287 | Ubiquitin-conjugating enzyme E2 N |
|  |  | PZE_104012758 | 4.02 | 10,877,840 | 8.23E-06 |  |  |
|  |  | SYN29169 | 2.04 | 37,932,091 | 1.16E-05 | GRMZM2G062488 | Acetylglucosaminyltransferase/ transferase |
|  |  | PZE_108044274 | 8.03 | 72,045,340 | 4.98E-05 |  |  |
|  |  | PZE_104130487 | 4.09 | 216,905,871 | 5.67E-05 | GRMZM2G104847 | Long chain acyl-CoA synthetase 2 |
|  |  | PZE_105102329 | 5.04 | 153,548,225 | 5.84E-05 |  |  |
|  |  | PZE_108028128 | 8.03 | 28,670,433 | 9.53E-05 | GRMZM2G451464 | DNA binding protein |
|  | QZ15 | SYN1153 | 5.05 | 179,071,638 | 1.80E-06 | GRMZM2G057184 | ARM-repeat/Tetratricopeptide repeat (TPR)-like protein |
|  |  | PZE_101116177 | 1.05 | 138,524,367 | 3.86E-06 | GRMZM2G014872 | SEC1 family transport protein SLY1 |
|  |  | SYN21847 | 5.07 | 209,066,383 | 2.81E-05 | GRMZM2G404762 | Galactoside 2-alpha-L-fucosyltransferase |
|  |  | PZE_101223850 | 1.10 | 273,945,146 | 6.16E-05 | GRMZM2G018798 | E3 ubiquitin protein ligase DRIP2 |
|  | QZ16 | PZE_110044605 | 9.03 | 84,917,062 | 1.29E-10 | GRMZM2G126007 | UPF0481 protein At3g47200 |
|  |  | SYN13476 | 1.08 | 248,406,024 | 4.11E-08 | GRMZM2G103843 | Fructokinase-like 2, chloroplastic |
|  |  | PZE_110054698 | 9.04 | 104,141,152 | 1.42E-06 |  |  |
|  |  | PZE_106033760 | 6.01 | 77,658,770 | 3.04E-06 | GRMZM2G342243 | Late embryogenesis abundant protein group 2 |
|  |  | PZE_102126139 | 2.06 | 172,650,265 | 6.94E-06 | GRMZM2G082487 | Probable protein phosphatase 2C 68 |
|  |  | SYN294 | 1.12 | 299,322,468 | 9.83E-06 | GRMZM2G377487 | Rhodanese-like domain-containing protein 8, chloroplast |
|  |  | PZE_107055832 | 7.02 | 102,439,641 | 1.34E-05 | GRMZM2G074672 | Vacuolar iron transporter 1.2-like |
|  |  | PZE_106027177 | 6.01 | 63,787,842 | 2.29E-05 |  |  |
|  |  | PZE_103084891 | 3.05 | 136,844,735 | 2.60E-05 | LOC103652225 | XS domain protein |
|  |  | PZE_109038139 | 9.03 | 54,569,925 | 8.20E-05 | GRMZM2G033566 | Anther-specific proline-rich protein APG |
